# Associations of Change in Body Size With All-Cause and Cause-Specific Mortality Among Healthy Older Adults

**DOI:** 10.1001/jamanetworkopen.2023.7482

**Published:** 2023-04-10

**Authors:** Sultana Monira Hussain, Anne B. Newman, Lawrence J. Beilin, Andrew M. Tonkin, Robyn L. Woods, Johannes T. Neumann, Mark Nelson, Prudence R. Carr, Christopher M. Reid, Alice Owen, Jocasta Ball, Flavia M. Cicuttini, Cammie Tran, Yuanyuan Wang, Michael E. Ernst, John J. McNeil

**Affiliations:** 1 School of Public Health and Preventive Medicine, Monash University, Melbourne, Victoria, Australia; 2 Department of Medical Education, Melbourne Medical School, The University of Melbourne, Melbourne, Victoria, Australia; 3 Center for Aging and Population Health, Department of Epidemiology, University of Pittsburgh, Pittsburgh, Pennsylvania; 4 Medical School, Royal Perth Hospital, University of Western Australia, Perth, Western Australia, Australia; 5 Department of Cardiology, University Heart & Vascular Center Hamburg, Hamburg, Germany; 6 German Center for Cardiovascular Research, Partner Site Hamburg/Kiel/Lübeck, Hamburg, Germany; 7 Discipline of General Practice, University of Tasmania, Hobart, Australia; 8 Department of Pharmacy Practice and Science, College of Pharmacy, The University of Iowa, Iowa City; 9 Department of Family Medicine, Carver College of Medicine, The University of Iowa, Iowa City

## Abstract

**Importance:**

The association between weight change and subsequent cause-specific mortality among older adults is not well described. The significance of changes in waist circumference (WC) has also not been compared with weight change for this purpose.

**Objective:**

To examine the associations of changes in body weight and WC with all-cause and cause-specific mortality.

**Design, Setting, and Participants:**

This cohort study is a post hoc analysis of data from the Aspirin in Reducing Events in the Elderly (ASPREE) randomized clinical trial, which recruited participants between March 1, 2010, and December 31, 2014. The study included community-based older adults (16 703 Australian participants aged ≥70 years and 2411 US participants aged ≥65 years) without evident cardiovascular disease (CVD), dementia, physical disability, or life-limiting chronic illness. Data analysis was performed from April to September 2022.

**Exposures:**

Body weight and WC were measured at baseline and at annual visit 2. Analysis models were adjusted for baseline body mass index because height and weight were measured at baseline, allowing for calculation of body mass index and other variables. Both body weight and WC changes were categorized as change within 5% (stable), decrease by 5% to 10%, decrease by more than 10%, increase by 5% to 10%, and increase by more than 10%.

**Main Outcomes and Measures:**

All-cause, cancer-specific, CVD-specific, and noncancer non-CVD–specific mortality. Mortality events were adjudicated by an expert review panel. Cox proportional hazards regression and competing risk analyses were used to calculate hazard ratios (HRs) and 95% CIs.

**Results:**

Among 16 523 participants (mean [SD] age, 75.0 [4.3] years; 9193 women [55.6%]), 1256 deaths were observed over a mean (SD) of 4.4 (1.7) years. Compared with men with stable weight, those with a 5% to 10% weight loss had a 33% higher (HR, 1.33; 95% CI, 1.07-1.66) risk of all-cause mortality, and those with more than a 10% decrease in body weight had a 289% higher (HR, 3.89; 95% CI, 2.93-5.18) risk. Compared with women with stable weight, those with a 5% to 10% weight loss had a 26% higher (HR, 1.26; 95% CI, 1.00-1.60) risk of all-cause mortality, and those with more than a 10% decrease in body weight had a 114% higher (HR, 2.14; 95% CI, 1.58-2.91) risk. Weight loss was associated with a higher cancer-specific mortality (>10% decrease among men: HR, 3.49; 95% CI, 2.26-5.40; 5%-10% decrease among women: HR, 1.44; 95% CI, 1.46-2.04; >10% decrease among women: HR, 2.78; 95% CI, 1.82-4.26), CVD-specific mortality (>10% decrease among men: HR, 3.14; 95% CI, 1.63-6.04; >10% decrease among women: HR, 1.92; 95% CI, 1.05-3.51), and noncancer non-CVD–specific mortality (>10% decrease among men: HR, 4.98; 95% CI, 3.14-7.91). A decrease in WC was also associated with mortality.

**Conclusions and Relevance:**

This cohort study of healthy older adults suggests that weight loss was associated with an increase in all-cause and cause-specific mortality, including an increased risk of cancer, CVD, and other life-limiting conditions. Physicians should be aware of the significance of weight loss, especially among older men.

## Key Points

**Question:**

Is change in body size associated with increased mortality risk among healthy older adults?

**Findings:**

In this cohort study of 16 523 community-dwelling healthy participants, 1256 died over a mean (SD) of 4.4 (1.7) years of follow-up. Among men, loss of 5% to 10% of body weight and loss of more than 10% of body weight were associated with a 33% and 289% increase in mortality, respectively; among women, loss of 5% to 10% of body weight and loss of more than 10% of body weight were associated with a 26% and 114% increase in mortality, respectively.

**Meaning:**

This study suggests that weight loss was associated with an increase in mortality, particularly among men, highlighting the need to monitor and investigate weight loss in older adults.

## Teaser

This cohort study uses data from the Aspirin in Reducing Events in the Elderly clinical trial to examine the associations of changes in body weight and waist circumference with all-cause and cause-specific mortality.

## Introduction

As individuals age, many experience a slow but progressive decrease in weight.^1^ In contrast, weight gain in others may accompany the adoption of a more sedentary existence.^2^ Physicians generally monitor weight changes in their older patients and target their advice, focusing particularly on the management of an idealized healthy weight. However, to our knowledge, the clinical significance of weight change has not been well documented in a population of relatively healthy older individuals free of diagnosed life-limiting illnesses.

One recently published systematic review of 30 studies reported that in older adults, weight loss, weight gain, or weight fluctuation was associated with increased risk of all-cause mortality.^3^ However, the studies included in this review differed substantially, involving varying measures of body size (mostly self-reported) and variability in the rigor with which mortality events were assessed. Most of the studies included individuals with preexisting illness, such as cancer, dementia, and cardiovascular disease (CVD). Thus, the mortality risk associated with weight loss or weight gain among apparently healthy older men and women requires further study.

Waist circumference (WC) may be a better measure than body weight to estimate all-cause mortality, CVD mortality, and premature mortality because it captures the negative outcomes of abdominal adiposity,^4^ which may be associated with an enhanced release of inflammatory mediators.^5^ However, data on the relative significance of changes in weight, WC, and cause-specific mortality have not been established in an aging population, to our knowledge.

In the Aspirin in Reducing Events in the Elderly (ASPREE) randomized clinical trial,^6^ body size parameters (weight and WC) were measured annually for a large population of initially healthy individuals. We used this information to investigate the associations of percentage change in body weight and WC with the risk of all-cause and cause-specific mortality in this population.

## Methods

### Study Design and Setting

The ASPREE trial recruited 19 114 community-dwelling adults from Australia (n = 16 703; aged ≥70 years) and the United States (n = 2411; aged ≥65 years) from March 1, 2010, to December 31, 2014. The detailed methods of the ASPREE trial have been previously reported.^6^ Participants were randomly assigned to receive 100 mg of aspirin daily or matching placebo.^6,7^ Participants were excluded if they had prior documented CVD events, dementia, physical disability, or a chronic illness expected to limit survival to less than 5 years. Ethics approval was obtained from institutional ethics review committees in Australia (the Royal Australasian College of General Practitioners Ethics Committee, the Monash Human Research Ethics Committee, the Human Research Ethics Committee [Tasmanian] Network, the Goulburn Valley Health Ethics & Research Committee, and ACT Health Human Research Ethics Committee) and the US (individual clinic sites were responsible for obtaining institutional review board approval from their respective institutions prior to study initiation). Participants provided written informed consent. This study followed the Strengthening the Reporting of Observational Studies in Epidemiology (STROBE) reporting guideline.

### Standard Assessments

After randomization, ASPREE trial participants were contacted quarterly by telephone and visited in person annually. At these annual visits, body weight, height at baseline, WC, and laboratory test measurements were recorded, and other health-related data were collected.^6^ Frailty was defined using the modified Fried frailty phenotype, as the presence of weakness, slowness, exhaustion, low physical activity, and a body mass index (BMI; calculated as weight in kilograms divided by height in meters squared) of less than 20 (included instead of weight loss).^7^ Any person with 1 or 2 of these criteria was categorized as prefrail, and those with 3 or more of these criteria were categorized as frail. Prespecified ASPREE end points (ie, cancer, clinically significant bleeding, dementia, depression, hospitalization for heart failure, myocardial infarction, and stroke) and hospitalizations were recorded at 6-month intervals by in-person interview or by a 6-month telephone call. All end point data were adjudicated by expert panels.^6^

### Assessment of Change in Body Size

We calculated percentage change in body weight from the difference in weight over the first 2 years after randomization as (weight at annual visit 2 − baseline weight) / baseline weight, expressed as a percentage. Change in WC was measured in a similar manner. The values for change in body-size indices (weight and WC) were divided into 5 categories: change within 5% (stable), decrease by 5% to 10%, decrease by more than 10%, increase by 5% to 10%, and increase by more than 10%.

### Assessment of Mortality

Mortality events were identified during routine follow-up by a review of health records when participants could not be contacted or when the next of kin or a close contact notified the trial center. In Australia, the trial staff performed weekly linkage with the Ryerson Index, a community-maintained register that monitors death notices and obituaries. The names of all Australian and US participants who had withdrawn or were lost to follow-up were linked to the National Death Index. Cause of death was established by an adjudication panel determining the single disease that was most likely to have initiated the trajectory toward death. Where relevant records could not be obtained, the underlying cause of death was based on *International Statistical Classification of Diseases and Related Health Problems, Tenth Revision* codes recorded on the death certificate or on the results of a search of the National Death Index. The present study analyzed change in body size between study entry and annual visit 2 and mortality that occurred after annual visit 2.

### Statistical Analysis

Statistical analysis was performed from April to September 2022. The characteristics of the participants were compared for continuous variables using analysis of variance and for categorical variables using χ^2^ tests, according to the weight change categories and WC change categories.

Cox proportional hazards regression was used to calculate the hazard ratios (HRs) and 95% CIs for all-cause mortality, and competing risk was used for cause-specific mortality. Initially, we examined the 5 weight change categories with all-cause mortality and cause-specific mortality using stable weight (within 5% change) as the reference category. Thereafter, we examined the 5 categories of WC change with mortality in a similar manner. All analyses were repeated in the same way for the individual end points of all-cause mortality, cancer-specific mortality, CVD-specific mortality, and noncancer non-CVD–specific mortality. Various analytical models were adjusted for 1 or more of the following characteristics: age, baseline BMI, baseline WC, frailty status, country of birth, smoking status, alcohol intake, educational level, hypertension, chronic kidney disease (estimated glomerular filtration rate, <60 mL/min/1.73 m^2^), diabetes, and interim hospitalization status.

There was a significant interaction between change in body size categories and sex that persisted in the fully adjusted models. There was no similar interaction between change in body size and age; analyses were therefore stratified by sex.

Sensitivity analyses were performed excluding the US population (predominantly comprising the US racial and ethnic minority population), excluding participants with a cognitive impairment at baseline,^8^ and restricting the analysis of outcomes to those occurring after annual visit 3 (to reduce the association with outcomes of illness progressing during years 1 and 2). Additional stratified analyses were performed based on baseline obesity status (nonobese BMI vs obese BMI), age category (<75 years vs ≥75 years), and the occurrence of hospitalization between baseline and annual visit 2, to account for individuals whose weight loss might have occurred during recovery after a hospitalization for an acute illness. Statistical significance was defined as a 2-sided *P* < .05. Stata MP, version 17 (StataCorp LLC), was used for analysis.

## Results

**Figure 1.**
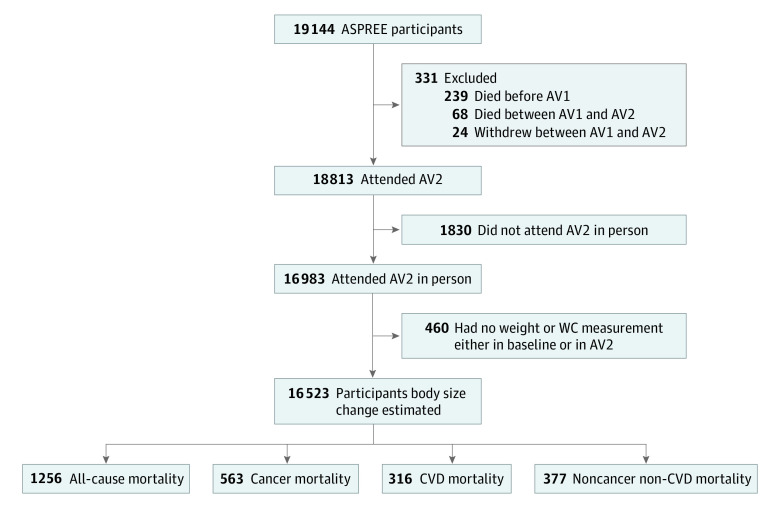
Percent Body Mass Index Changes and Mortality Ascertainment Timeline Among the Aspirin in Reducing Events in the Elderly (ASPREE) Trial Participants AV indicates annual visit; CVD, cardiovascular disease; and WC, waist circumference.

Of the 19 114 participants, 16 523 (mean [SD] age, 75.0 [4.3] years; 9193 women [55.6%] and 7330 men [44.4%]) had both weight and WC measured at baseline and annual visit 2, allowing for estimation of percentage change in body size (Figure 1). Among these participants, 1256 mortality events were recorded. The participants were followed up for a mean (SD) of 4.4 (1.7) years.

### Characteristic of Participants

The body weight of 12 370 participants (74.9%) remained within 5% (stable) during the first 2 years (baseline to annual visit 2), 1299 (7.9%) had a 5% to 10% increase in weight, and 314 (1.9%) had an increase of more than 10%. Conversely, 1953 participants (11.8%) had a 5% to 10% decrease in weight, and 587 (3.6%) had a decrease of more than 10% (Table 1). Those who had a decrease in weight were mainly women, from the US, and prefrail or frail.

**Table 1.** Baseline Characteristics of Aspirin in Reducing Events in the Elderly Trial Participants Who Remained Free of Mortality Through the Second Annual Visit

Characteristic	Participants, No. (%)						*P* value
	All	Within 5% weight change or stable weight	Increase in weight		Decrease in weight		
			5%-10%	>10%	5%-10%	>10%	
No. (%)	16 523 (100.0)	12 370 (74.9)	1299 (7.9)	314 (1.9)	1953 (11.8)	587 (3.6)	NA
Age, mean (SD), y	75.0 (4.3)	75.0 (4.4)	74.6 (4.1)	74.7 (4.5)	75.5 (4.8)	75.6 (4.8)	<.001
Sex							
Male	7330 (44.4)	5750 (46.5)	525 (40.4)	107 (34.1)	765 (39.2)	183 (31.2)	<.001
Female	9193 (55.6)	6620 (53.5)	774 (59.6)	207 (65.9)	1188 (60.8)	404 (68.8)	
Country							
Australia	14 632 (88.6)	10 984 (88.8)	1170 (90.1)	277 (88.2)	1711 (87.6)	490 (83.5)	<.001
US	1891 (11.4)	1386 (11.2)	129 (9.9)	37 (11.8)	242 (12.4)	97 (16.5)	
Low physical activity	1139 (6.9)	806 (6.5)	88 (6.8)	23 (7.3)	167 (8.6)	55 (9.4)	.002
Weight, mean (SD), kg	77.1 (14.8)	77.2 (14.6)	75.2 (14.5)	71.8 (14.2)	77.3 (15.2)	80.1 (16.9)	<.001
Waist circumference, mean (SD), cm	97.1 (12.7)	97.1 (12.5)	96.4 (13.2)	95.1 (13.0)	97.5 (13.2)	98.9 (14.4)	<.001
Current or former smoking	7276 (44.0)	5387 (43.5)	594 (45.7)	151 (48.1)	863 (44.2)	281 (47.9)	.08
Current alcohol use	12 806 (77.5)	9717 (78.6)	992 (76.4)	231 (73.6)	1453 (74.4)	413 (70.4)	<.001
Educational level, y							
<12	9371 (56.7)	6980 (56.4)	795 (61.2)	194 (61.8)	1083 (55.5)	319 (54.3)	.002
≥12	7152 (43.3)	5390 (43.6)	504 (38.8)	120 (38.2)	870 (44.5)	268 (45.7)	
Hypertension	12 222 (74.0)	9104 (73.6)	956 (73.6)	225 (71.7)	1492 (76.4)	445 (75.8)	.07
Dyslipidemia	10 789 (65.3)	8040 (65.0)	846 (65.1)	207 (65.9)	1321 (67.6)	375 (63.9)	.21
Chronic kidney disease	2878/16 113 (17.9)	2099/12 067 (17.4)	214/1265 (16.9)	59/305 (19.3)	384/1905 (20.2)	122/571 (21.4)	.005
Diabetes	1721 (10.4)	1236 (10.0)	135 (10.4)	41 (13.1)	245 (12.5)	64 (10.9)	.006
Prefrail or frail	6503 (39.4)	4708 (38.1)	509 (39.2)	145 (46.2)	861 (44.1)	280 (47.7)	<.001
≥1 Interim hospitalization	3403 (20.6)	2356 (19.0)	276 (21.2)	80 (25.5)	476 (24.4)	215 (36.6)	<.001
Taking trial medication (100 mg of aspirin)	8199 (49.6)	6125 (49.5)	651 (50.1)	163 (51.9)	961 (49.2)	299 (50.9)	.85
Outcomes							
All-cause mortality	1256 (7.6)	850 (6.9)	87 (6.7)	30 (9.6)	183 (9.4)	106 (18.1)	<.001
Cancer mortality	563/15 830 (3.6)	385/11 905 (3.2)	39/1251 (3.1)	11/295 (3.7)	79/1849 (4.3)	49/530 (9.2)	<.001
Cardiovascular disease mortality	316/15 583 (2.0)	215/11 735 (1.8)	25/1237 (2.0)	6/290 (2.1)	47/1817 (2.6)	23/504 (4.6)	<.001
Noncancer non-CVD mortality	377/15 644 (2.4)	250/11 770 (2.1)	23/1235 (1.9)	13/297 (4.4)	57/1827 (3.1)	34/515 (6.6)	<.001

Abbreviations: CVD, cardiovascular disease; NA, not applicable.

The characteristics of participants according to change in WC are shown in eTable 1 in Supplement 1. Of the included participants, 10 234 (61.9%) had a stable WC (within 5% change), 295 (12.7%) had a 5% to 10% increase, and 973 (5.9%) had an increase in their WC of more than 10%. Conversely, 2309 participants (14.0%) had a decrease of 5% to 10%, and 912 (5.5%) had a decrease of more than 10% in their WC. Those with a decrease of more than 10% in WC were mainly US participants, women, prefrail or frail, and those with impaired kidney function.

### Change in Body Weight and Mortality

#### All-Cause Mortality

**Figure 2.**
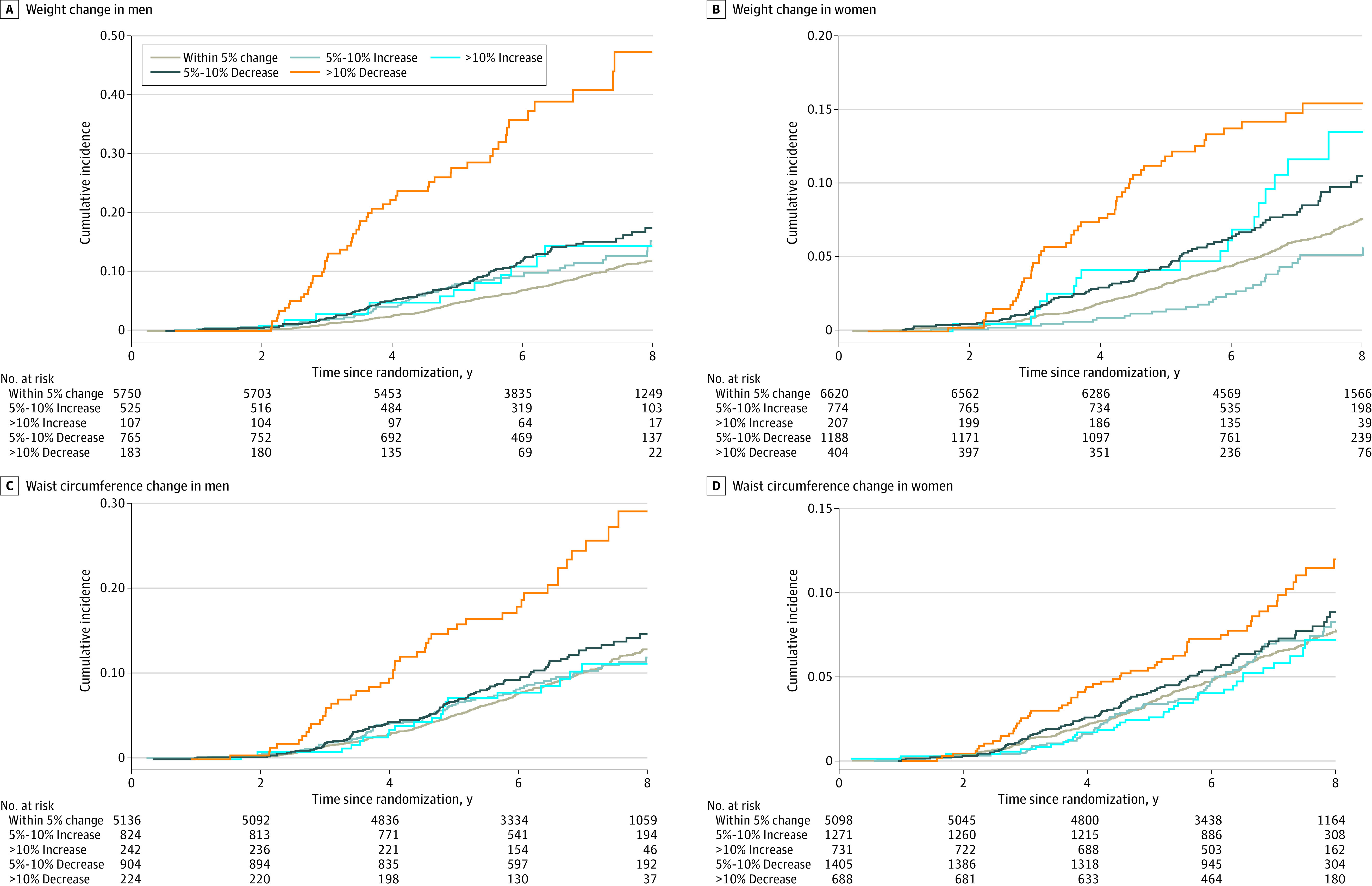
Associations of Changes in Body Size and All-Cause Mortality in the Aspirin in Reducing Events in the Elderly Trial

Table 2 shows the association between percentage change in BMI and subsequent all-cause and cause-specific mortality. The lowest risk of all-cause mortality was observed for the stable weight group (Figure 2). A total of 484 of mortality events were recorded among 5750 men with stable weight (8.4%), while 55 mortality events were recorded among 183 men with more than 10% weight loss (30.1%) (Table 2). Compared with men who had a stable weight (change within 5%), those who had a 5% to 10% decrease in weight had a 33% higher risk of all-cause mortality (HR, 1.33; 95% CI, 1.07-1.66), and those who had a more than 10% decrease in weight had a 289% higher (HR, 3.89; 95% CI, 2.93-5.18) risk. Hazard ratios were only modestly changed after adjustment for age, frailty status, country of birth, smoking status, alcohol intake, educational level, hypertension, chronic kidney disease, diabetes, and interim hospitalization. A decrease in weight was also associated with a higher mortality risk among women (5%-10% decrease: HR, 1.26; 95% CI, 1.00-1.60; >10% decrease: HR, 2.14; 95% CI, 1.58-2.91). A total of 366 mortality events were recorded among 6620 women with stable weight (5.5%), while 51 mortality events were recorded among 404 women with more than 10% weight loss (12.6%).

**Table 2.** Association Between Body Weight Change Categories and Risk of Mortality in Men and Women^a^

Type of mortality	Hazard ratio (95% CI)				
	Within 5% of weight change or stable weight	Increase in weight		Decrease in weight	
		5%-10%	>10%	5%-10%	>10%
**All-cause mortality**					
Men					
Events, No./total No. (%)	484/5750 (8.4)	56/525 (10.7)	12/107 (11.2)	97/765 (12.7)	55/183 (30.1)
Risk/10 000 person-years	128	168	179	201	555
Model 1	1 [Reference]	1.44 (1.09-1.90)	1.56 (0.88-2.75)	1.42 (1.14-1.76)	4.72 (3.56-6.23)
Model 2	1 [Reference]	1.45 (1.10-1.92)	1.59 (0.89-2.82)	1.41 (1.13-1.75)	4.68 (3.53-6.18)
Model 3	1 [Reference]	1.46 (1.11-1.93)	1.54 (0.87-2.72)	1.38 (1.11-1.72)	4.56 (3.44-6.03)
Model 4	1 [Reference]	1.45 (1.09-1.91)	1.37 (0.75-2.49)	1.35 (1.08-1.68)	4.23 (3.19-5.61)
Model 5	1 [Reference]	1.45 (1.10-1.92)	1.38 (0.76-2.52)	1.33 (1.07-1.66)	3.89 (2.93-5.18)
Women					
Events, No./total No. (%)	366/6620 (5.5)	31/774 (4.0)	18/207 (8.7)	86/1188 (7.2)	51/404 (12.6)
Risk per 10 000 person-years	83	60	137	112	204
Model 1	1 [Reference]	0.78 (0.54-1.13)	1.67 (1.04-2.68)	1.30 (1.03-1.65)	2.46 (1.83-3.29)
Model 2	1 [Reference]	0.79 (0.55-1.13)	1.70 (1.06-2.73)	1.29 (1.02-1.64)	2.41 (1.80-3.24)
Model 3	1 [Reference]	0.77 (0.53-1.11)	1.61 (1.01-1.62)	1.28 (1.01-1.63)	2.37 (1.77-3.19)
Model 4	1 [Reference]	0.75 (0.52-1.09)	1.49 (0.91-2.42)	1.27 (1.01-1.62)	2.24 (1.66-3.03)
Model 5	1 [Reference]	0.75 (0.52-1.09)	1.47 (0.90-2.39)	1.26 (1.00-1.60)	2.14 (1.58-2.91)
**Cancer mortality**					
Men					
Events, No./total No. (%)	227/5493 (4.1)	25/494 (5.1)	6/101 (5.9)	39/707 (5.5)	23/151 (15.2)
Risk per 10 000 person-years	60	75	89	81	232
Model 1	1 [Reference]	1.35 (0.89-2.04)	1.62 (0.72-3.67)	1.26 (0.89-1.77)	4.27 (2.78-6.56)
Model 2	1 [Reference]	1.36 (0.91-2.07)	1.75 (0.78-3.94)	1.22 (0.87-1.72)	4.07 (2.64-6.26)
Model 3	1 [Reference]	1.36 (0.90-2.05)	1.62 (0.72-3.64)	1.24 (0.88-1.74)	4.20 (2.73-6.45)
Model 4	1 [Reference]	1.36 (0.90-2.06)	1.58 (0.70-3.55)	1.23 (0.87-1.73)	3.91 (2.54-6.04)
Model 5	1 [Reference]	1.37 (0.91-2.08)	1.60 (0.71-3.60)	1.20 (0.85-1.69)	3.49 (2.26-5.40)
Women					
Events, No./total No. (%)	158/6412 (2.5)	14/757 (1.8)	5/194 (2.6)	40/1142 (3.5)	26/379 (6.9)
Risk per 10 000 person-years	36	27	38	53	104
Model 1	1 [Reference]	0.79 (0.45-1.36)	1.07 (0.44-2.62)	1.44 (1.02-2.03)	2.97 (1.96-4.50)
Model 2	1 [Reference]	0.79 (0.46-1.37)	1.10 (0.45-2.68)	1.42 (1.01-2.01)	2.87 (1.89 to 4.36)
Model 3	1 [Reference]	0.78 (0.45-1.35)	1.06 (0.43-2.57)	1.43 (1.01-2.02)	2.93 (1.94-4.44)
Model 4	1 [Reference]	0.79 (0.46-1.37)	0.81 (0.30-2.18)	1.46 (1.03-2.06)	2.90 (1.90-4.44)
Model 5	1 [Reference]	0.79 (0.46-1.36)	0. 80 (0.30-2.15)	1.44 (1.02-2.04)	2.78 (1.82-4.26)
**CVD mortality**					
Men					
Events, No./total No. (%)	115/5381 (2.1)	15/484 (3.1)	2/97 (2.1)	25/693 (3.6)	10/138 (7.2)
Risk per 10 000 person-years	30	45	30	51	101
Model 1	1 [Reference]	1.67 (0.98-2.87)	1.12 (0.28-4.52)	1.49 (0.97-2.30)	3.69 (1.93-7.04)
Model 2	1 [Reference]	1.68 (0.98-2.88)	1.14 (0.28-4.60)	1.48 (0.96-2.28)	3.64 (1.90-6.98)
Model 3	1 [Reference]	1.70 (0.99-2.91)	1.10 (0.27-4.47)	1.45 (0.94-2.24)	3.56 (1.86-6.80)
Model 4	1 [Reference]	1.58 (0.90-2.75)	1.10 (0.27-4.48)	1.34 (0.86-2.10)	3.30 (1.72-6.35)
Model 5	1 [Reference]	1.58 (0.90-2.78)	1.11 (0.27-4.32)	1.38 (0.89-2.13)	3.14 (1.63-6.04)
Women					
Events, No./total No. (%)	100/6354 (1.6)	10/753 (1.3)	4/193 (2.1)	22/1124 (2.0)	13/366 (3.6)
Risk per 10 000 person-years	23	19	30	29	52
Model 1	1 [Reference]	0.95 (0.50-1.82)	1.37 (0.51-3.72)	1.20 (0.76-1.91)	2.25 (1.26-4.02)
Model 2	1 [Reference]	0.97 (0.51-1.86)	1.44 (0.53-3.91)	1.18 (0.74-1.87)	2.11 (1.18-3.77)
Model 3	1 [Reference]	0.94 (0.49-1.79)	1.33 (0.49-3.61)	1.18 (0.75-1.88)	2.19 (1.23- 3.90)
Model 4	1 [Reference]	0.93 (0.48-1.78)	1.32 (0.48-3.58)	1.15 (0.72-1.84)	2.01 (1.10-3.68)
Model 5	1 [Reference]	0.93 (0.48-1.78)	1.30 (0.48-3.54)	1.14 (0.71-1.82)	1.92 (1.05-3.51)
**Noncancer non-CVD mortality**					
Men					
Events, No./total No. (%)	142/5408 (2.6)	16/485 (3.3)	4/99 (4.0)	33/701 (4.7)	22/150 (14.7)
Risk per 10 000 person-years	38	48	59	69	222
Model 1	1 [Reference]	1.42 (0.85-2.38)	1.78 (0.66-4.81)	1.59 (1.09-2.33)	6.21 (3.96-9.74)
Model 2	1 [Reference]	1.41 (0.84-2.37)	1.66 (0.61-4.51)	1.64 (1.13-2.41)	6.49 (4.13-10.21)
Model 3	1 [Reference]	1.46 (0.87-2.44)	1.75 (0.65-4.73)	1.52 (1.04-2.23)	5.83 (3.71-9.16)
Model 4	1 [Reference]	1.48 (0.88-2.49)	1.24 (0.39-3.89)	1.53 (1.04-2.24)	5.39 (3.41-8.52)
Model 5	1 [Reference]	1.48 (0.88-2.50)	1.25 (0.40-3.94)	1.51 (1.03-2.21)	4.98 (3.14-7.91)
Women					
Events, No./total No. (%)	108/6362 (1.7)	7/750 (0.9)	9/198 (4.5)	24/1126 (2.1)	12/365 (3.3)
Risk per 10 000 person-years	25	14	68	32	48
Model 1	1 [Reference]	0.62 (0.29-1.34)	2.85 (1.44-5.62	1.21 (0.77-1.88)	1.91 (1.05-3.46)
Model 2	1 [Reference]	0.61 (0.28-1.31)	2.74 (1.39-5.43)	1.22 (0.78-1.89)	1.98 (1.09-3.60)
Model 3	1 [Reference]	0.60 (0.28-1.29)	2.63 (1.34-5.20)	1.17 (0.75-1.81)	1.77 (0.98-3.22)
Model 4	1 [Reference]	0.52 (0.23-1.19)	2.60 (1.31-5.15)	1.13 (0.72-1.78)	1.56 (0.84-2.91)
Model 5	1 [Reference]	0.52 (0.23-1.19)	2.58 (1.30-5.11)	1.12 (0.71-1.76)	1.49 (0.80-2.79)

Abbreviation: CVD, cardiovascular disease.

^a^ There was a significant interaction between the direction of the weight change and sex (*P* < .001); therefore, results are presented by sex. Models adjusted for age (model 1); model 1 plus baseline body mass index (model 2); model 1 plus frailty status (model 3); model 3 plus country of birth, smoking status, alcohol intake, educational level, hypertension, chronic kidney disease, and diabetes (model 4); and model 4 plus interim hospitalization (model 5).

#### Cancer Mortality

In the fully adjusted analysis, a decrease of more than 10% in weight was associated with a 3.5-fold (HR, 3.49; 95% CI, 2.26-5.40) higher risk of cancer-specific mortality among men (Table 2). A decrease of 5% or more in weight was associated with cancer mortality among women (5%-10% decrease: HR, 1.44; 95% CI, 1.02-2.04; >10% decrease: HR, 2.78; 95% CI, 1.82-4.26). Increase in weight was not associated with cancer mortality.

#### CVD Mortality

A more than 10% decrease in weight was associated with higher CVD mortality for both men (HR, 3.14; 95% CI, 1.63-6.04) and women (HR, 1.92; 95% CI, 1.05-3.51) (Table 2). Increase in weight was not associated with CVD mortality.

#### Noncancer Non-CVD Mortality

A 10% or more decrease in weight was associated with a 5-fold higher risk of noncancer non-CVD mortality for men (HR, 4.98; 95% CI, 3.14-7.91) (Table 2). However, this association was not seen for women (HR, 1.49; 95% CI, 0.80-2.79).

### Change in WC and Mortality

The associations of change in WC with all-cause and cause-specific mortality are presented in Table 3. A more than 10% decrease in WC was associated with 2.14-fold higher (HR, 2.14; 95% CI, 1.57-2.91) risk in all-cause mortality for men and a 34% higher (HR, 1.34; 95% CI, 1.02-1.78) risk in all-cause mortality for women. There was no association between a 5% to 10% decrease in WC or any increase in WC and all-cause mortality (Figure 2).

**Table 3.** Association Between WC Change Categories and Risk of Mortality in Men and Women^a^

Type of mortality	Hazard ratio (95% CI)				
	Within 5% of WC change	Increase in WC		Decrease in WC	
		5%-10%	>10%	5%-10%	>10%
**All-cause mortality**					
Men					
Events, No./total No. (%)	463/5136 (9.0)	77/824 (9.3)	21/242 (8.7)	98/904 (10.8)	45/224 (20.1)
Risk per 10 000 person-years	139	142	135	168	324
Model 1	1 [Reference]	1.07 (0.85-1.36)	0.96 (0.62-1.48)	1.15 (0.93-1.44)	2.24 (1.65-3.04)
Model 2	1 [Reference]	1.12 (0.88-1.43)	1.05 (0.67-1.62)	1.14 (0.92-1.42)	2.27 (1.67-3.09)
Model 3	1 [Reference]	1.08 (0.85-1.38)	0.94 (0.61-1.46)	1.15 (0.92-1.43)	2.15 (1.58-2.29)
Model 4	1 [Reference]	1.10 (0.87-1. 40)	0.90 (0.57-1.42)	1.13 (0.90-1.40)	2.18 (1.61-2.97)
Model 5	1 [Reference]	1.11 (0.88-1.42)	0.90 (0.57-1.42)	1.10 (0.89-1.38)	2.14 (1.57-2.91)
Women					
Events, No./total No. (%)	286/5098 (5.6)	77/1271 (6.1)	37/731 (5.1)	91/1405 (6.5)	61/688 (8.9)
Risk per 10 000 person-years	85	91	77	99	134
Model 1	1 [Reference]	1.06 (0.83-1.36)	0.89 (0.64-1.26)	1.13 (0.90-1.43)	1.42 (1.07-1.87)
Model 2	1 [Reference]	1.08 (0.84-1.39)	0.96 (0.68-1.37)	1.12 (0.88-1.42)	1.37 (1.03-1.80)
Model 3	1 [Reference]	1.07 (0.83-1.37)	0.89 (0.63-1.25)	1.14 (0.90-1.44)	1.37 (1.04-1.81)
Model 4	1 [Reference]	1.07 (0.83-1.39)	0.88 (0.62-1.24)	1.17 (0.93-1.49)	1.35 (1.03-1.79)
Model 5	1 [Reference]	1.07 (0.83-1.38)	0.87 (0.62-1.24)	1.16 (0.92-1.48)	1.34 (1.02-1.78)
**Cancer mortality**					
Men					
Events, No./total No. (%)	215/4888 (4.4)	42/789 (5.3)	9/230 (3.9)	36/842 (4.3)	18/197 (9.1)
Risk per 10 000 person-years	65	77	58	61	130
Model 1	1 [Reference]	1.23 (0.88-1.71)	0.89 (0.46-1.73)	0.92 (0.65-1.31)	1.98 (1.23-3.21)
Model 2	1 [Reference]	1.33 (0.95-1.85)	1.04 (0.53-2.03)	0.90 (0.63-1.28)	2.02 (1.25-3.27)
Model 3	1 [Reference]	1.23 (0.89-1.72)	0.88 (0.45-1.72)	0.93 (0.64-1.31)	1.94 (1.20-3.14)
Model 4	1 [Reference]	1.24 (0.89-1.74)	0.83 (0.41-1.67)	0.90 (0.64-1.29)	1.93 (1.20-3.13)
Model 5	1 [Reference]	1.25 (0.89-1.75)	0.82 (0.40-1.66)	0.88 (0.62-1.26)	1.87 (1.15-3.03)
Women					
Events, No./total No. (%)	124/4936 (2.5)	32/1226 (2.6)	17/711 (2.4)	44/1358 (3.2)	26/653 (4.0)
Risk per 10 000 person-years	37	38	35	48	57
Model 1	1 [Reference]	1.01 (0.69-1.50)	0.95 (0.56-1.57)	1.28 (0.91-1.80)	1.44 (0.95-2.20)
Model 2	1 [Reference]	1.05 (0.71-1.54)	1.02 (0.61-1.69)	1.26 (0.89-1.77)	1.38 (0.90-2.11)
Model 3	1 [Reference]	1.02 (0.69-1.50)	0.94 (0.57-1.56)	1.28 (0.91-1.81)	1.42 (0.93-2.17)
Model 4	1 [Reference]	1.05 (0.71-1.55)	0.88 (0.51-1.49)	1.33 (0.95-1.88)	1.50 (0.98-2.29)
Model 5	1 [Reference]	1.05 (0.71-1.54)	0.88 (0.51-1.40)	1.32 (0.94-1.87)	1.49 (0.97-2.28)
**CVD mortality**					
Men					
Events, No./total No. (%)	114/4787 (2.4)	12/759 (1.6)	6/227 (2.6)	26/832 (3.1)	9/188 (4.8)
Risk per 10 000 person-years	34	22	39	45	65
Model 1	1 [Reference]	0.70 (0.38-1.25)	1.10 (0.48-2.49)	1.24 (0.81-1.90)	1.77 (0.90-3.50)
Model 2	1 [Reference]	0.72 (0.40-1.31)	1.19 (0.52-2.73)	1.23 (0.80-1.88)	1.80 (0.91-3.56)
Model 3	1 [Reference]	0.70 (0.40-1.27)	1.08 (0.48-2.45)	1.24 (0.81-1.89)	1.71 (0.87-3.37)
Model 4	1 [Reference]	0.71 (0.39-1.29)	0.92 (0.38-2.25)	1.20 (0.78-1.84)	1.72 (0.87-3.35)
Model 5	1 [Reference]	0.71 (0.39-1.28)	0.92 (0.38-2.26)	1.19 (0.77-1.82)	1.71 (0.87-3.38)
Women					
Events, No./total No. (%)	76/4888 (1.6)	24/1218 (2.0)	10/704 (1.4)	23/1337 (1.7)	16/643 (2.5)
Risk per 10 000 person-years	23	28	21	25	35
Model 1	1 [Reference]	1.23 (0.78-1.95)	0.91 (0.47-1.77)	1.08 (0.67-1.72)	1.36 (0.80-2.34)
Model 2	1 [Reference]	1.29 (0.81-2.04)	1.02 (0.52-1.99)	1.05 (0.66-1.68)	1.27 (0.73-2.18)
Model 3	1 [Reference]	1.25 (0.79-1.97)	0.91 (0.47-1.76)	1.09 (0.68-1.73)	1.33 (0.77-2.27)
Model 4	1 [Reference]	1.32 (0.83-2.09)	0.94 (0.49-1.82)	1.15 (0.72-1.83)	1.17 (0.66-2.08)
Model 5	1 [Reference]	1.32 (0.82-2. 08)	0.94 (0.49-1.82)	1.13 (0.70-1.81)	1.15 (0.65-2.05)
**Noncancer non-CVD mortality**					
Men					
Events, No./total No. (%)	134/4807 (2.8)	23/770 (3.0)	6/227 (2.6)	36/842 (4.3)	18/197 (9.1)
Risk per 10 000 person-years	40	42	39	62	130
Model 1	1 [Reference]	1.13 (0.73-1.76)	0.94 (0.42-2.10)	1.46 (1.01-2.10)	3.03 (1.85-4.95)
Model 2	1 [Reference]	1.12 (0.72-1.76)	0.93 (0.41-2.14)	1.46 (1.01-2.11)	3.02 (1.84-4.94)
Model 3	1 [Reference]	1.14 (0.74-1.78)	0.92 (0.41-2.08)	1.44 (1.00-2.09)	2.83 (1.72-4.64)
Model 4	1 [Reference]	1.20 (0.77-1.87)	0.98 (0.43-2.22)	1,42 (0.98-2.06)	2.99 (1.82-4.91)
Model 5	1 [Reference]	1.21 (0.77-1.89)	0.98 (0.43-2.22)	1.39 (0.96-2.02)	2.93 (1.78-4.80)
Women					
Events, No./total No. (%)	86/4898 (1.8)	21/1215 (1.7)	10/704 (1.4)	24/1338 (1.8)	19/646 (2.9)
Risk per 10 000 person-years	26	25	20	26	42
Model 1	1 [Reference]	0.95 (0.59-1.53)	0.81 (0.42-1.55)	0.98 (0.63-1.54)	1.44 (0.88-2.37)
Model 2	1 [Reference]	0.95 (0.59-1.53)	0.80 (0.41-1.56)	0.99 (0.63-1.55)	1.45 (0.88-2.39)
Model 3	1 [Reference]	0.98 (0.61-1.57)	0.80 (0.41-1.57)	1.00 (0.64-1.57)	1.35 (0.82-2.23)
Model 4	1 [Reference]	0.90 (0.55-1.48)	0.82 (0.42-1.57)	0.99 (0.62-1.57)	1.33 (0.81-2.20)
Model 5	1 [Reference]	0.90 (0.54-1.48)	0.82 (0.42-1.55)	0.98 (0.62-1.55)	1.32 (0.80-2.17)

Abbreviations: CVD, cardiovascular; WC, waist circumference.

^a^ There was a significant interaction between the direction of the WC change and sex (*P* = .01); therefore, results are presented by sex. Models adjusted for age, (model 1); model 1 plus baseline WC (model 2); model 1 plus frailty status (model 3); model 3 plus country of birth, smoking status, alcohol intake, educational level, hypertension, chronic kidney disease, and diabetes (model 4); and model 4 plus interim hospitalization (model 5).

A more than 10% decrease in WC was associated with higher cancer mortality for men (HR, 1.87; 95% CI, 1.15-3.03) and women (HR, 1.49; 95% CI, 0.97-2.28) in fully adjusted analyses (Table 3). Change in WC was not associated with CVD mortality. A more than 10% decrease in WC was associated with higher noncancer non-CVD mortality among men (HR, 2.93; 95% CI, 1.78-4.80) only. Change in WC was not associated with noncancer non-CVD mortality among women.

### Sensitivity Analyses

The associations persisted when we excluded participants from the US and participants who had any evidence of cognitive impairment at baseline and after restricting the analysis to outcomes occurring after annual visit 3 (eTables 2, 3, and 4 in Supplement 1). Similar associations of change in body weight with all-cause mortality and cause-specific mortality were observed among participants with or without obesity, as well as participants who were younger than 75 years at recruitment or those 75 years or older at recruitment (eTable 5 and eTable 6 in Supplement 1). The associations of change in BMI with all-cause and cause-specific mortality also remained similar when the analyses were stratified by interim hospitalization status (eTable 7 in Supplement 1). The associations of change in WC with all-cause mortality and cause-specific mortality remained similar to our main analyses in all the sensitivity analyses (eTables 8-13 in Supplement 1).

## Discussion

The principal finding of this study was that a weight loss of more than 10% was associated with higher all-cause mortality (including increased mortality from cancer, CVD, and noncancer non-CVD) among both men and women. The association was more pronounced among men, for whom, in absolute terms, subsequent all-cause mortality was 8.4% among those maintaining consistent weight over a mean (SD) of 4.4 (1.7) years vs 30.1% among those for whom weight decreased by more than 10%; for women, the equivalent mortality rates were 5.5% and 12.6%. Lesser weight loss (between 5% and 10%) was associated with higher all-cause mortality for both sexes compared with stable weight, as well as a higher cancer mortality for women and a higher noncancer non-CVD mortality for men.

A decrease in WC was also associated with increased mortality. Weight gain, however, was not significantly associated with mortality, with the possible exception of noncancer non-CVD mortality for women. However, based on the small number of events in that category, this result should be interpreted cautiously.

Previous studies have reported an association between weight loss and subsequent mortality, but these studies included only a small number of older adults, typically with multiple comorbidities (eTable 14 in Supplement 1).^3,9,10,11,12,13,14,15,16,17,18,19,20,21,22^ Our study extends the previous observations by demonstrating a similar association among relatively healthy community-dwelling individuals aged 65 years or older. The results also showed that weight loss was more associated with mortality among men than women.

Two previous longitudinal studies examined the association of weight loss with CVD and cancer mortality and yielded conflicting results. The Enquête de Santé Psychologique-Risques, Incidence et Traitement (ESPRIT) study showed that weight loss was associated with higher CVD mortality but not cancer mortality.^22^ By contrast, the Guangzhou Biobank study showed that self-reported weight loss was associated with higher CVD mortality and cancer mortality.^13^ These studies differed from the present cohort by including participants with various chronic diseases and not adjusting for or excluding individuals with a recent hospitalization.^13,22^ Our study showed that weight loss was associated with all-cause mortality and an increase in all major causes of death, including cancer and CVD in an initially healthy population. In addition, our study showed that these results persisted even after adjustment for age, frailty status, baseline BMI, country of birth, smoking, hypertension, diabetes, and hospitalization in the previous 24 months. Adjustment for recent hospitalization is important because hospitalization is often followed by weight loss due to acute conditions.

Our study demonstrates that weight loss in older men and women is associated with an increased mortality risk irrespective of an individual’s baseline weight (ie, unanticipated weight loss even among adults with obesity is associated with increased mortality, regardless of other potential benefits of weight loss that are associated with quality of life and other morbidities). In addition, our study has clarified the lesser implications of weight gain. Compared with previous reports, the present study is based on individual measures of weight (rather than self-report) and adjudicated assessments of the cause of death. Without adjudication, deaths among older populations may be misclassified in up to 30% cases.^23^

The observation that weight loss was associated with mortality among men may be the result of the different body composition characteristics of men and women.^24^ For men, a higher proportion of body mass is constituted of muscle and bone mass, whereas for women, a higher proportion of body mass is composed of fat.^24^ If weight loss preceding chronic illness is predominantly loss of muscle mass and bone mass, it could explain the differences observed between men and women. Something similar might be at work to explain why weight loss, rather than decrease of WC, is more associated with mortality.

A likely explanation for these findings is that weight loss can be an early prodromal indicator of the presence of various life-shortening diseases.^25,26^ Although it is widely acknowledged that weight loss may precede a diagnosis of cancer, in our study, weight loss also preceded an increased mortality from CVD and other causes. The latter may include deaths from trauma, dementia, Parkinson disease, and other less common causes.^27^

In this age group, weight loss was largely associated with a reduction of appetite, leading to reduced food intake. Appetite is a complex process, governed by both the central nervous system and various circulating hormones. Several proposals have been put forward to explain why appetite might be suppressed in the early stages of chronic illness, ranging from increases in resistance to appetite-stimulating hormones,^28^ increased levels of inflammatory cytokines,^29^ and high levels of other mediators such as growth differentiation factor 15.^30^ Plasma concentrations of the latter are increased in chronic inflammatory disease, most subtypes of cancer, and cardiovascular and kidney disease, and several longitudinal studies have shown it to be independently associated with decreases in muscle mass and muscle strength.^30^

### Strengths and Limitations

This study has some strengths. The combination of the large cohort, the focus on the population older than 70 years, and the extensive amount of hospitalization data, coupled with the regular and objective measures of body size and expert adjudication of cause of death, makes this the most comprehensive and detailed study of weight loss in this age group yet published, to our knowledge. It is coherent with but extends the findings of a meta-analysis whose component studies typically involved a broader range of ages, recall of prior weight, and/or reliance on death certificate data for cause of death.^31^

This study also has some limitations. The principal limitation is the inability to differentiate intended vs unintended weight loss, although bariatric surgery, the only likely intervention proven capable of intended long-term sustained weight loss,^32^ is rarely undertaken in this age group. Furthermore, exploring whether change in activity level and diet quality between baseline and annual visit 2 had any association with outcomes was not possible because they were not recorded in this study. Residual confounding, such as intended weight loss or change in activity or diet, cannot be excluded.

## Conclusions

The clinical implication of the findings of this cohort study is that physicians should be aware of the significant association with mortality of even relatively minor weight loss (≥5%), especially among older men. The risk extends beyond an increased risk of cancer, extending to CVD and a range of other life-limiting conditions. Further research will be needed to determine more precisely the association between weight loss and the onset of fatal diseases and whether clinical or laboratory investigations can identify individuals for whom early intervention may be effective.

## Notes

Supplement 1. **eTable 1.** Characteristics of Aspirin in Reducing Events in the Elderly Trial Participants Overall, and by Waist Circumference (WC) Change Category From Recruitment Until Annual Visit 2 **eTable 2.** Association Between Weight Change Categories and Risk of Mortality (HR, 95% CI) Excluding the US Participants (6500 Men and 7704 Women) **eTable 3.** Association Between Body Weight Change Categories and Risk of Mortality (HR, 95% CI) Excluding Those With Baseline Cognitive Impairment (7128 Men, 8984 Women) **eTable 4.** Association Between Body Weight Change Categories and Risk of Mortality (HR, 95% CI) (Restricting the Analysis After AV3: 6161 Men, 7817 Women) **eTable 5.** Association Between Body Weight Change Categories and Risk of Mortality (HR, 95% CI) Stratified by Obesity Status **eTable 6.** Association Between Body Weight Change Categories and Risk of Mortality (HR, 95% CI) Stratified by Age at Recruitment **eTable 7.** Association Between Body Weight Change Categories and Risk of Mortality (HR, 95% CI) Stratified by Interim Hospitalisation Status During Change in Body Size Measurement **eTable 8.** Association Between Waist Circumference Change Categories and Risk of Mortality (HR, 95% CI) Excluding the US **eTable 9.** Association Between Waist Circumference Change Categories and Risk of Mortality (HR, 95% CI) Excluding Those With Baseline Cognitive Impairment **eTable 10.** Association Between Waist Circumference Change Categories and Risk of Mortality (HR, 95% CI) (Restricting the Analysis After AV3) **eTable 11.** Association Between Waist Circumference Change Categories and Risk of Mortality (HR, 95% CI) Stratified by Obesity Status **eTable 12.** Association Between Waist Circumference Change Categories and Risk of Mortality (HR, 95% CI) Stratified by Age at Recruitment **eTable 13.** Association Between Waist Circumference Change Categories and Risk of Mortality (HR, 95% CI) Stratified by Interim Hospitalisation Status **eTable 14.** Overview of Systematic Reviews and Cohort Studies Investigating the Association Between Weight Change in Kilogram, Body Mass Index, Waist Circumference and Mortality in Noninstitutionalized People

Supplement 2.
Data Sharing Statement
