# Supplementary material for: Associations of Change in Body Size With All-Cause and Cause-Specific Mortality Among Healthy Older Adults
Source: JAMA Netw Open. 2023 Apr 10;6(4):e237482. doi: 10.1001/jamanetworkopen.2023.7482 (PMC10087052; doi:10.1001/jamanetworkopen.2023.7482)
Supplement: Supplement 1. — eTable 1. Characteristics of Aspirin in Reducing Events in the Elderly Trial Participants Overall, and by Waist Circumference (WC) Change Category From Recruitment Until Annual Visit 2 eTable 2. Association Between Weight Change Categories and Risk of Mortality (HR, 95% CI) Excluding the US Participants (6500 Men and 7704 Women) eTable 3. Association Between Body Weight Change Categories and Risk of Mortality (HR, 95% CI) Excluding Those With Baseline Cognitive Impairment (7128 Men, 8984 Women) eTable 4. Association Between Body Weight Change Categories and Risk of Mortality (HR, 95% CI) (Restricting the Analysis After AV3: 6161 Men, 7817 Women) eTable 5. Association Between Body Weight Change Categories and Risk of Mortality (HR, 95% CI) Stratified by Obesity Status eTable 6. Association Between Body Weight Change Categories and Risk of Mortality (HR, 95% CI) Stratified by Age at Recruitment eTable 7. Association Between Body Weight Change Categories and Risk of Mortality (HR, 95% CI) Stratified by Interim Hospitalisation Status During Change in Body Size Measurement eTable 8. Association Between Waist Circumference Change Categories and Risk of Mortality (HR, 95% CI) Excluding the US eTable 9. Association Between Waist Circumference Change Categories and Risk of Mortality (HR, 95% CI) Excluding Those With Baseline Cognitive Impairment eTable 10. Association Between Waist Circumference Change Categories and Risk of Mortality (HR, 95% CI) (Restricting the Analysis After AV3) eTable 11. Association Between Waist Circumference Change Categories and Risk of Mortality (HR, 95% CI) Stratified by Obesity Status eTable 12. Association Between Waist Circumference Change Categories and Risk of Mortality (HR, 95% CI) Stratified by Age at Recruitment eTable 13. Association Between Waist Circumference Change Categories and Risk of Mortality (HR, 95% CI) Stratified by Interim Hospitalisation Status eTable 14. Overview of Systematic Reviews and Cohort Studies Investigating the As [file jamanetwopen-e237482-s001.pdf]

Supplementary Online Content

Hussain SM, Newman AB, Beilin LJ, et al. Associations of change in body size with all-cause and cause-specific mortality among healthy older adults. *JAMA Netw Open.* 2023;6(4):e237482. doi:10.1001/jamanetworkopen.2023.7482

eTable 1. Characteristics of Aspirin in Reducing Events in the Elderly Trial Participants Overall, and by Waist Circumference (WC) Change Category From Recruitment Until Annual Visit 2
eTable 2. Association Between Weight Change Categories and Risk of Mortality (HR, 95% CI) Excluding the US Participants (6500 Men and 7704 Women)
eTable 3. Association Between Body Weight Change Categories and Risk of Mortality (HR, 95% CI) Excluding Those With Baseline Cognitive Impairment (7128 Men, 8984 Women)
eTable 4. Association Between Body Weight Change Categories and Risk of Mortality (HR, 95% CI) (Restricting the Analysis After AV3: 6161 Men, 7817 Women)
eTable 5. Association Between Body Weight Change Categories and Risk of Mortality (HR, 95% CI) Stratified by Obesity Status
eTable 6. Association Between Body Weight Change Categories and Risk of Mortality (HR, 95% CI) Stratified by Age at Recruitment
eTable 7. Association Between Body Weight Change Categories and Risk of Mortality (HR, 95% CI) Stratified by Interim Hospitalisation Status During Change in Body Size Measurement
eTable 8. Association Between Waist Circumference Change Categories and Risk of Mortality (HR, 95% CI) Excluding the US
eTable 9. Association Between Waist Circumference Change Categories and Risk of Mortality (HR, 95% CI) Excluding Those With Baseline Cognitive Impairment
eTable 10. Association Between Waist Circumference Change Categories and Risk of Mortality (HR, 95% CI) (Restricting the Analysis After AV3)
eTable 11. Association Between Waist Circumference Change Categories and Risk of Mortality (HR, 95% CI) Stratified by Obesity Status
eTable 12. Association Between Waist Circumference Change Categories and Risk of Mortality (HR, 95% CI) Stratified by Age at Recruitment
eTable 13. Association Between Waist Circumference Change Categories and Risk of Mortality (HR, 95% CI) Stratified by Interim Hospitalisation Status
eTable 14. Overview of Systematic Reviews and Cohort Studies Investigating the Association Between Weight Change in Kilogram, Body Mass Index, Waist Circumference and Mortality in Noninstitutionalized People

This supplementary material has been provided by the authors to give readers additional information about their work.

© 2023 Hussain SM et al. *JAMA Network Open.*

eTable 1. Characteristics of Aspirin in Reducing Events in the Elderly Trial Participants Overall, and by Waist Circumference (WC) Change Category From Recruitment Until Annual Visit 2

	All	Within 5% change	5-10% increase	>10% increase	5-10% decrease	>10% decrease	p
N (%)	16523	10234 (61.9)	295 (12.7)	973 (5.9)	2309 (14.0)	912 (5.5)	
Age in years, mean (SD)	75.0 (4.4)	75.0 (4.4)	74.8 (4.3)	75.0 (4.4)	75.1 (4.6)	75.7 (4.5)	<.001
Women, n (%)	9193 (55.6)	5098 (49.8)	1271 (60.7)	731 (75.2)	1405 (60.9)	688 (75.4)	<.001
Country, n (%)							<.001
Australia	14632 (88.6)	9152 (89.4)	1845 (88.1)	814 (83.7)	2041 (88.4)	780 (85.5)	
USA	1891 (11.4)	1082 (10.6)	250 (11.9)	159 (16.3)	268 (11.6)	132 (14.5)	
Low physical activity, n (%)	1139 (6.9)	682 (6.7)	146 (7.0)	65 (6.7)	171 (7.4)	75 (8.2)	.38
BMI (kg/m2), mean (SD)	28.1 (4.6)	28.2 (4.6)	27.8 (4.6)	27.4 (4.8)	28.2 (4.9)	27.8 (5.0)	<.001
Waist Circumference (cm), mean (SD)	97.1 (12.7)	98.2 (12.4)	93.2 (11.6)	87.2 (11.9)	99.1 (12.7)	99.4 (13.0)	<.001
Current/former smoking, n (%)	7276 (44.0)	4615 (45.1)	900 (43.0)	397 (40.1)	982 (42.5)	382 (42.0)	.01
Current alcohol use, n (%)	12806 (77.5)	8014 (78.3)	1625 (77.6)	743 (76.4)	1761 (76.3)	663 (72.7)	.001
Education, n (%)							.05
<12 years of schooling	9371 (56.7)	5877 (57.4)	1179 (56.3)	521 (53.6)	1303 (56.4)	491 (53.8)	
≥12 years of schooling	7152 (43.3)	4357 (42.6)	916 (43.7)	452 (46.5)	1006 (43.6)	421 (46.2)	
Hypertension, (%)	12222 (74.0)	7618 (74.4)	1496 (71.4)	711 (73.1)	1724 (74.7)	673 (73.8)	.06
Dyslipidaemia, (%)	10789 (65.3)	6681 (65.3)	1349 (64.4)	624 (64.1)	1527 (66.1)	608 (66.7)	.56
Chronic kidney disease, (%)	2878 (17.9)	1746 (17.5)	334 (16.3)	183 (19.3)	417 (18.5)	200 (22.3)	.001
Diabetes, n (%)	1721 (10.4)	1126 (11.0)	197 (9.4)	79 (8.1)	248 (10.7)	71 (7.8)	.001
Prefrail/Frail, (%)	6503 (39.4)	3928 (38.4)	803 (38.3)	414 (42.6)	940 (40.7)	418 (45.8)	<.001
≥1 Interim hospitalisation	3403 (20.6)	2052 (20.1)	407 (19.4)	198 (20.4)	517 (22.4)	229 (25.1)	<.001
On trial medication (100mg Aspirin), (%)	8199 (49.6)	5029 (49.1)	1042 (49.7)	516 (53.0)	1177 (51.0)	435 (47.7)	.07
**Outcome**							
All-cause mortality, (%)	1256 (7.6)	749 (7.3)	154 (7.4)	58 (6.0)	189 (8.2)	106 (11.6)	<.001
Cancer Mortality, (%)	563 (3.6)	339 (3.5)	74 (3.7)	26 (2.8)	80 (3.6)	44 (5.2)	.04
Cardiovascular disease mortality, (%)	316 (2.0)	190 (2.0)	36 (1.8)	16 (1.7)	49 (2.3)	25 (3.0)	.24
Non-CV, non-Cancer mortality	377 (2.4)	220 (2.3)	44 (2.2)	16 (1.7)	60 (2.8)	37 (4.4)	.001

SD, Standard deviation; USA, United States of America; WC, waist circumference; CV, Cardiovascular

© 2023 Hussain SM et al. *JAMA Network Open.*

eTable 2. Association Between Weight Change Categories and Risk of Mortality (HR, 95% CI) Excluding the US Participants (6500 Men and 7704 Women)

	Within 5% of change	5-10% increase	>10% increase	5-10% decrease	>10% decrease
All-cause mortality					
Men	1	1.53 (1.15-2.03)	1.52 (0.83-2.77)	1.30 (1.04-1.65)	4.02 (3.00-5.39)
Women	1	0.65 (0.43, 1.00)	1.33 (0.78-2.28)	1.31 (1.02-1.70)	2.10 (1.51-2.94)
Cancer death					
Men	1	1.46 (0.97, 2.22)	1.74 (0.77-3.93)	1.16 (0.81-1.66)	3.67 (2.35-5.63)
Women	1	0.86 (0.49, 1.-48)	0.86 (0.32, 2.32)	1.49 (1.04, 2.14)	2.50 (1.56-4.00)
Cardiovascular disease mortality					
Men	1	1.70 (0.97-2.00)	1.24 (0.30, 5.03)	1.28 (0.80, 2.06)	3.52 (1.82-6.82)
Women	1	0.60 (0.26-1.38)	1.40 (0.51, 3.83)	1.12 (0.68, 1.86)	1.83 (0.95, 3.55)
Non cancer, non CV mortality					
Men	1	1.48 (0.87-2.54)	1.38 (0.44-4.34)	1.53 (1.03-2.28)	4.91 (3.02-7.98)
Women	1	0.32 (0.11-1.01)	2.07 (0.90-4.74)	1.21 (0.74-2.00)	1.76 (0.91-3.43)

US, United States; Adjusted for age, frailty status, smoking status, alcohol intake, education, hypertension, chronic kidney disease, diabetes, interim hospitalisation status

© 2023 Hussain SM et al. *JAMA Network Open.*

eTable 3. Association Between Body Weight Change Categories and Risk of Mortality (HR, 95% CI) Excluding Those With Baseline Cognitive Impairment (7128 Men, 8984 Women)

	Within 5% change	5-10% increase	>10% increase	5-10% decrease	>10% decrease
All-cause mortality					
Men	1	1.44 (1.09-1.91)	1.37 (0.75-2.49)	1.35 (1.08-1.69)	4.23 (3.19-5.61)
Women	1	0.75 (0.52-1.09)	1.47 (0.90-2.39)	1.27 (0.99- 1.60)	2.14 (1.58-2.90)
Cancer death					
Men	1	1.36 (0.89-2.06)	1.58 (0.70-3.59)	1.23 (0.89-1.73)	3.92 (2.54-6.03)
Women	1	0.79 (0.45-1.36)	0.80 (0.30-2.15)	1.44 (1.01-2.03)	2.78 (1.82-4.26)
Cardiovascular mortality					
Men	1	1.58 (0.90-2.75)	1.10 (0.27-4.48)	1.35 (0.86, 2.10)	3.31 (1.71-6.35)
Women	1	0.93 (0.48-1.78)	1.30 (0.48-3. 54)	1.14 (0.71-1.82)	1.92 (1.05-3.51)
Non cancer, non CV mortality					
Men	1	1.48 (0.88-2.49)	1.24 (0.39-3.90)	1.53 (1.04-2.24)	5.39 (3.41-8.52)
Women	1	0.52 (0.23-1.19)	2.58 (1.30-5.11)	1.12 (0.71-1.76)	1.49 (0.80-2.79)

Adjusted for age, frailty status, country of birth, smoking status, alcohol intake, education, hypertension, chronic kidney disease, diabetes, interim hospitalisation status

© 2023 Hussain SM et al. *JAMA Network Open.*

eTable 4. Association Between Body Weight Change Categories and Risk of Mortality (HR, 95% CI) (Restricting the Analysis After AV3: 6161 Men, 7817 Women)

	Within 5% change	5-10% increase	>10% increase	5-10% decrease	>10% decrease
All-cause mortality					
Men	1	1.40 (1.02-1.92)	1.25 (0.62-2.52)	1.31 (1.02-1.68)	3.09 (2.13-4.48)
Women	1	0.84 (0.56-1.24)	1.51 (0.86-2.63)	1.20 (0.90-1.58)	1.63 (1.08-2.46)
Cancer death					
Men	1	1.62 (1.06-2.48)	1.30 (0.48-3.51)	1.29 (0.89-1.86)	2.03 (1.07-3.87)
Women	1	0.90 (0.51-1.58)	1.01 (0.37-2.75)	1.28 (0.85-1.93)	1.95 (1.09-3.46)
Cardiovascular disease mortality					
Men	1	1.43 (0.74-2.74)	1.39 (0.34-5.65)	1.40 (0.86-2.27)	3.18 (1.46-6.93)
Women	1	0.97 (0.47-2.01)	1.30 (0.41-4.12)	1.10 (0.63-1.92)	1.42 (0.62-3.29)
Non cancer, non CV mortality					
Men	1	0.96 (0.47-2.00)	1.06 (0.26-4.39)	1.24 (0.78-1.98)	4.80 (2.73, 8.54)
Women	1	0.59 (0.24-1.45)	2.52 (1.07-5.79)	1.15 (0.67-1.98)	1.34 (0.58-3.08)

AV3, Annual visit 3; Adjusted for age, frailty status, country of birth, smoking status, alcohol intake, education, hypertension, chronic kidney disease, diabetes, interim hospitalisation status

© 2023 Hussain SM et al. *JAMA Network Open.*

eTable 5. Association Between Body Weight Change Categories and Risk of Mortality (HR, 95% CI) Stratified by Obesity Status

	Non-obese (n=11669)					Obese (n=4832)				
	Within 5% change	5-10% increase	>10% increase	5-10% decrease	>10% decrease	Within 5% change	5-10% increase	>10% increase	5-10% decrease	>10% decrease
All-cause mortality										
Men	1	1.48 (1.09-2.06)	1.230 (0.63-2.39)	1.23 (0.94-1.61)	4.29 (3.04-6.05)	1	1.34 (0.76-2.39)	2.60 (0.64-10.60)	1.58 (1.072.34)	4.20 (2.52-6.97)
Women	1	0.72 (0.46-1.13)	1.17 (0.63-2.22)	1.37 (1.06-1.82)	2.19 (1.47-3.27)	1	0.83 (0.42-1.63)	2.49 (1.16-5.37)	1.09 (0.70-1.70)	2.29 (1.42-3.70)
Cancer death										
Men	1	1.32 (0.80-2.13)	1.49 (0.60-3.63)	1.01 (0.65-1.57)	4.04 (2.37-6.90)	1	1.48 (0.67-3.20)	2.34 (0.32-17.40)	1.57 (0.92-2.71)	3.81 (1.79-8.13)
Women	1	0.77 (0.40-1.47)	1.08 (0.40-2.95)	1.64 (1.09-2.41)	2.44 (1.34-4.44)	1	0.81 (0.29-2.24)	-	1.18 (0.61-2.28)	3.55 (1.90-6.63)
Cardiovascular disease mortality										
Men	1	1.70 (0.91-3.21)	1.24 (0.314.99)	1.35 (0.83-2.27)	2.46 (1.00-6.15)	1	1.20 (0.36-3.98)	-	1.25 (0.54-2.92)	4.53 (1.71-12.0)
Women	1	0.91 (0.41-1.89)	0.91 (0.22-3.75)	0.98 (0.54-1.78)	1.99 (0.91-4.35)	1	1.02 (0.31-3.39)	2.76 (0.65-11.66)	1.50 (0.71-3.18)	2.04 (0.78-5.32)
Non cancer, non CV mortality										
Men	1	1.58 (0.90-2.81)	0.87 (0.21-3.45)	1.44 (0.91-2.26)	5.93 (3.54-9.9)	1	1.26 (0.38-4.17)	6.46 (0.85-49.8)	1.94 (0.92-4.07)	4.49 (1.70-11.82)
Women	1	0.47 (0.17-1.27)	1.56 (0.57-4.31)	1.41 (0.84-2.33)	2.04 (0.98-4.25)	1	0.70 (0.17-2.92)	5.46 (2.10-14.2)	0.59 (0.21-1.68)	0.98 (0.29-3.24)

Adjusted for age, frailty status, country of birth, smoking status, alcohol intake, education, hypertension, chronic kidney disease, diabetes, interim hospitalisation status

© 2023 Hussain SM et al. *JAMA Network Open.*

eTable 6. Association Between Body Weight Change Categories and Risk of Mortality (HR, 95% CI) Stratified by Age at Recruitment

	<75 years (n=9922)					>=75 years (n=6851)				
	Within 5% change	5-10% increase	>10% increase	5-10% decrease	>10% decrease	Within 5% change	5-10% increase	>10% increase	5-10% decrease	>10% decrease
All-cause mortality										
Men	1	1.17 (0.73-1.87)	1.35 (0.55-3.29)	1.40 (0.96-2.06)	4.85 (2.97-7.92)	1	1.63 (1.15-2.30)	1.39 (0.62-3.12)	1.34 (1.04-1.74)	4.26 (3.01-6.04)
Women	1	0.80 (0.44-1.47)	1.01 (0.37-2.86)	1.46 (1.012.20)	2.40 (1.40-4.12)	1	0.72 (0.45-1.17)	1.70 (0.97-2.98)	1.18 (0.89-1.58)	2.15 (1.46-3.10)
Cancer death										
Men	1	1.12 (0.55-2.20)	2.10 (0.77-5.56)	1.48 (0.90-2.56)	4.59 (2.30-9.16)	1	1.59 (0.95-2.69)	1.10 (0.27-4.49)	1.13 (0.73-1.75)	3.70 (2.11-6.45)
Women	1	0.58 (0.23-1.44)	-	1.69 (1.02-2.81)	3.18 (1.68-6.04)	1	0.98 (0.49-1.95)	1.42 (0.51-3.83)	1.27 (0.80-2.05)	2.64 (1.50-4.65)
Cardiovascular disease mortality										
Men	1	1.53 (0.59-3.92)	1.28 (0.17-9.04)	1.43-(0.60-3.43)	4.21 (1.29-13.84)	1	1.51 (0.75-3.01)	0.85 (0.12-6.15)	1.28 (0.76-2.15)	3.07 (1.38-6.74)
Women	1	1. 86 (0.69-4.93)	1.52 (0.20-11.0)	1.42 (0.53-3.67)	1.76 (0.41-7.64)	1	0.62 (0.25-1.54)	1.26 (0.40-3.99)	1.11 (0.66-1.88)	2.07 (1.07-4.01)
Non cancer, non CV mortality										
Men	1	1.04 (0.42-2.61)	-	1.21 (0.57-2.55)	5.27 (2.24-12.40)	1	1.79 (0.96-3.38)	2.33 (0.73-7.39)	1.68 (1.07-2.63)	6.15 (3.55-10.67)
Women	1	0.55 (0.13-2.30)	2.82 (0.85-9.34)	0.95 (0.36-2.48)	1.18 (0.28-5.01)	1	0.49 (0.18-1.35)	2.47 (1.07-5.42)	1.16 (0.70-1.94)	1.66 (0.83-3.33)

Adjusted for age, frailty status, country of birth, smoking status, alcohol intake, education, hypertension, chronic kidney disease, diabetes, interim hospitalisation status

© 2023 Hussain SM et al. *JAMA Network Open.*

eTable 7. Association Between Body Weight Change Categories and Risk of Mortality (HR, 95% CI) Stratified by Interim Hospitalisation Status During Change in Body Size Measurement

	Not hospitalised between baseline and annual visit 2 (n=13120)					Hospitalised before annual visit 2 (n=3403)				
	Within 5% change	5-10% increase	>10% increase	5-10% decrease	>10% decrease	Within 5% change	5-10% increase	>10% increase	5-10% decrease	>10% decrease
All-cause mortality										
Men	1	1.47 (1.04-2.01)	1.36 (0.67-2.75)	1.18 (0.90-1.58)	3.18 (2.06-4.91)	1	1.48 (0.86-2.53)	1.40 (0.45-4.54)	1.60 (1.11-2.31)	4.60 (3.21-6.80)
Women	1	0.57 (0.36-0.91)	1.03 (0.53-2.03)	1.26 (0.98-1.67)	1.53 (1.00-2.39)	1	1.40 (0.76-2.60)	2.61 (1.22-5.49)	1.28 (0.79-2.11)	3.58 (2. 28-5.61)
Cancer death										
Men	1	1.37 (0.83-2.23)	1.49 (0.55-4.02)	1.00 (0.63-1.56)	2.25 (1.06-4.73)	1	1.37 (0.62-3.00)	1.91 (0.46-7.85)	1.59 (0.94-2.69)	4.71 (2.71-8.20)
Women	1	0.72 (0.38-1.36)	0.78 (0.25-2.24)	1.42 (0.98-2.11)	1. 74 (0.98-3.28)	1	1.01 (0.36-2.90)	0.78 (0.12-5.72)	1.79 (0.89, 3.57)	6.06 (3.25, 11.30)
Cardiovascular disease mortality										
Men	1	1.99 (1.11-3.58)	0.73 (0.10-5.27)	1.34 (0.79-2.26)	3.04 (1.22-7.56)	1	0.46 (0.07-3.38)	2.01 (0.28-15.81)	1.26 (0.54-2.91)	3,32 (1.26-8.77)
Women	1	0.60 (0.24-1.47)	0.43 (0.06-3.07)	1.35 (0.82-2.23)	1.40 (0.57-3.46)	1	2.01 (0. 78-5.61)	3.43 (0.98-5.61)	0.61 (0.18-2.05)	2.87 (1.19-6.87)
Non cancer, non CV mortality										
Men	1	1.12 (0.582.22)	1.65 (0.525.24)	1.36 (0.84-2.21)	4.71 (2.43-9.12)	1	2.65 (1.17, 6.01)	-	1.90 (1.00-3.59)	5.43 (2.77-10.63)
Women	1	0.31 (0.10-1.01)	1.86 (0.75, 4. 59)	0.95 (0.55, 1.66)	1.34 (0.58, 3.08)	1	1.37 (0.41-4.66)	4.88 (1.62-14.73)	1.64 (0.72-3.72)	2.05 (0.76-5.52)

Adjusted for age, frailty status, country of birth, smoking status, alcohol intake, education, hypertension, chronic kidney disease, diabetes

© 2023 Hussain SM et al. *JAMA Network Open.*

eTable 8. Association Between Waist Circumference Change Categories and Risk of Mortality (HR, 95% CI) Excluding the US

	Within 5% change	5-10% increase	>10% increase	5-10% decrease	>10% decrease
All-cause mortality					
Men	1	1.17 (0.91-1.51)	0.93 (0.58-1.50)	1.19 (0.95-1.49)	2.24 (1.62-3.09)
Women	1	0.65 (0.43-1.00)	1.34 (0.78-2.29)	1.32 (1.03-1.71)	2.21 (1.59-3.07)

US, United States; WC, waist circumference; Adjusted for age, frailty status, smoking status, alcohol intake, education, hypertension, chronic kidney disease, diabetes, interim hospitalisation status

© 2023 Hussain SM et al. *JAMA Network Open.*

eTable 9. Association Between Waist Circumference Change Categories and Risk of Mortality (HR, 95% CI) Excluding Those With Baseline Cognitive Impairment

	Within 5% change	5-10% increase	>10% increase	5-10% decrease	>10% decrease
All-cause mortality					
Men	1	1.10 (0.86-1.40)	0.90 (0.57-1.42)	1.13 (0.91-1.41)	2.18 (1.61-2.98)
Women	1	1.07 (0.83-1.39)	0.87 (0.61-1.23)	1.17 (0.93-1.48)	1.35 (1.02, 1.79)

WC, waist circumference; Adjusted for age, frailty status, country of birth, smoking status, alcohol intake, education, hypertension, chronic kidney disease, diabetes, interim hospitalisation status

© 2023 Hussain SM et al. *JAMA Network Open.*

eTable 10. Association Between Waist Circumference Change Categories and Risk of Mortality (HR, 95% CI) (Restricting the Analysis After AV3)

	Within 5% change	5-10% increase	>10% increase	5-10% decrease	>10% decrease
All-cause mortality					
Men	1	1.07 (0.81-1.40)	0.77 (0.44-1.34)	1.10 (0.86-1.40)	1.76 (1.21, 2.58)
Women	1	1.18 (0.89-1.56)	1.03 (0.70-1.50)	1.06 (0.79-1.39)	1.28 (0.92-1.80)

AV3, annual visit 3; WC, waist circumference; Adjusted for age, frailty status, country of birth, smoking status, alcohol intake, education, hypertension, chronic kidney disease, diabetes, interim hospitalisation status

© 2023 Hussain SM et al. *JAMA Network Open.*

eTable 11. Association Between Waist Circumference Change Categories and Risk of Mortality (HR, 95% CI) Stratified by Obesity Status

	Non-obese (n=11669)					Obese (n=4832)				
	Within 5% change	5-10% increase	>10% increase	5-10% decrease	>10% decrease	Within 5% change	5-10% increase	>10% increase	5-10% decrease	>10% decrease
All-cause mortality										
Men	1	1.13 (0.85-1.50)	0.96 (0.58-1.59)	1.14 (0.89-1.48)	2.37 (1.69-3.32)	1	1.01 (0.62-1.66)	0.70 (0.22-2.20)	1.09 (0.70-1.69)	1.54 (0.71-3.31)
Women	1	1.13 (0.83-1.53)	0.95 (0.64-1.43)	1.22 (0.91-1.62)	1.53 (1.10-2.11)	1	0.97 (0.61-1.55)	0.69 (0.32-1.49)	1.11 (0.73-1.68)	1.00 (0.56-1.79)

WC, waist circumference; Adjusted for age, frailty status, country of birth, smoking status, alcohol intake, education, hypertension, chronic kidney disease, diabetes, interim hospitalisation status

© 2023 Hussain SM et al. *JAMA Network Open.*

eTable 12. Association Between Waist Circumference Change Categories and Risk of Mortality (HR, 95% CI) Stratified by Age at Recruitment

	<75 years (n=9783)					>=75 years (n=6740)				
	Within 5% change	5-10% increase	>10% increase	5-10% decrease	>10% decrease	Within 5% change	5-10% increase	>10% increase	5-10% decrease	>10% decrease
All-cause mortality										
Men	1	1.16 (0.79-1.71)	0.53 (0.20-1.42)	1.03 (0.68-1.53)	2.40 (1.45-3.96)	1	1.05 (0.77-1.44)	1.08 (0.64-1.82)	1.19 (0.92-1.54)	2.05 (1.39-3.03)
Women	1	1.11 (0.72-1.69)	0.65 (0.33-1.30)	1.22 (0.81-1.83)	1.17 (0.65-2.08)	1	1.05 (0.78, 1.46)	0.97 (0.64, 1.82)	1.15 (0.86-1.54)	1.41 (1.02-1.95)

WC, waist circumference; Adjusted for age, frailty status, country of birth, smoking status, alcohol intake, education, hypertension, chronic kidney disease, diabetes, interim hospitalisation status

© 2023 Hussain SM et al. *JAMA Network Open.*

eTable 13. Association Between Waist Circumference Change Categories and Risk of Mortality (HR, 95% CI) Stratified by Interim Hospitalisation Status

	Not hospitalized before annual visit 2 (n=13120)					Hospitalised before annual visit 2 (n=3403)				
	Within 5% change	5-10% increase	>10% increase	5-10% decrease	>10% decrease	Within 5% change	5-10% increase	>10% increase	5-10% decrease	>10% decrease
All-cause mortality										
Men	1	1.19 (0.90-1.57)	0.87 (0.50-1.52)	0.91 (0.68-1.21)	1.68 (1.10-2.55)	1	0.89 (0.54-1.49)	0.94 (0. 42-2.15)	1. 52 (1.07-2.13)	3.00 (1.89-4.71)
Women	1	1.01 (0.75-1.36)	0.67 (0.43-1.04)	1.21 (0.92-1.04)	1.11 (0.78-1.57)	1	1.24 (0.76-2.04)	1.52 (0.85-2.74)	1.06 (0.65-1.74)	2.00 (1.22-3.23)

WC, waist circumference; Adjusted for age, frailty status, country of birth, smoking status, alcohol intake, education, hypertension, chronic kidney disease, diabetes

© 2023 Hussain SM et al. *JAMA Network Open.*

eTable 14. Overview of Systematic Reviews and Cohort Studies Investigating the Association Between Weight Change in Kilogram, Body Mass Index, Waist Circumference and Mortality in Noninstitutionalized People

Author, Year	Study population	Study name	Exposure	Findings related to mortality	Comments
Systematic reviews					
Alharbi 2021[3]	30 studies, including participants aged >65 years Studies published before June 2020 were included	N/A	Weight gain, weight loss, weight fluctuation	**All-cause mortality**: Based on 30 studies weight loss was associated with a 59% increase risk· Based on 27 studies weight gain was associated with a 10% increase risk·<br>Based on 4 studies weight fluctuation was associated with a 63% increased risk·	Only in 6 studies change in BMI was examined· In the rest of the studies change in weight (kg) was examined·<br><br>High heterogeneity between the included studies
Karahalios 2017[9]	26 studies including participants aged 40-65 years·<br>Studies were published before September 2015	N/A	Weight gain and weight loss	**All-cause mortality**: Weight loss increased the risk by 45%<br>Weight gain was not associated<br><br>**Cancer mortality**: neither weight loss nor weight gain was associated<br><br>**CVD mortality**: Weight gain increased the risk by 21%· Weight loss was not associated	High heterogeneity between the included studies<br><br>Most studies examined weight change and, in a few studies, change in BMI was examined·
Cohort studies not included in the two systematic reviews					
Alharbi 2022[22]	1664 community-dwelling adults aged 65 years	Enquête de Santé Psychologique-Risques,	Weight change between 1999–2000 and 2003–2004	**Cancer Mortality**: ≥5% weight loss was not associated with cancer mortality	Participants had several comorbidities i·e· 32% participants had depression

© 2023 Hussain SM et al. *JAMA Network Open.*

Author, Year	Study population	Study name	Exposure	Findings related to mortality	Comments
Systematic reviews					
Alharbi 2021[3]	30 studies, including participants aged >65 years Studies published before June 2020 were included	N/A	Weight gain, weight loss, weight fluctuation	**All-cause mortality**: Based on 30 studies weight loss was associated with a 59% increase risk· Based on 27 studies weight gain was associated with a 10% increase risk·<br>Based on 4 studies weight fluctuation was associated with a 63% increased risk·	Only in 6 studies change in BMI was examined· In the rest of the studies change in weight (kg) was examined·<br><br>High heterogeneity between the included studies
Karahalios 2017[9]	26 studies including participants aged 40-65 years·<br>Studies were published before September 2015	N/A	Weight gain and weight loss	**All-cause mortality**: Weight loss increased the risk by 45%<br>Weight gain was not associated<br><br>**Cancer mortality**: neither weight loss nor weight gain was associated<br><br>**CVD mortality**: Weight gain increased the risk by 21%· Weight loss was not associated	High heterogeneity between the included studies<br><br>Most studies examined weight change and, in a few studies, change in BMI was examined·
		Incidence et Traitement (ESPRIT) study		**CVD mortality**: ≥5% weight loss was associated with higher CVD mortality (HR: 1·53, 95% CI: 1·10–2·14)	
Alharbi 2022[21]	2,017 community-	Enquête de Santé Psychologique-	Self-reported weight loss during	**All-cause mortality**: Self-reported weight loss at baseline was not associated	Participants had several comorbidities i·e· one in three had depression

© 2023 Hussain SM et al. *JAMA Network Open.*

Author, Year	Study population	Study name	Exposure	Findings related to mortality	Comments
Systematic reviews					
Alharbi 2021[3]	30 studies, including participants aged >65 years Studies published before June 2020 were included	N/A	Weight gain, weight loss, weight fluctuation	**All-cause mortality**: Based on 30 studies weight loss was associated with a 59% increase risk· Based on 27 studies weight gain was associated with a 10% increase risk·<br>Based on 4 studies weight fluctuation was associated with a 63% increased risk·	Only in 6 studies change in BMI was examined· In the rest of the studies change in weight (kg) was examined·<br><br>High heterogeneity between the included studies
Karahalios 2017[9]	26 studies including participants aged 40-65 years·<br>Studies were published before September 2015	N/A	Weight gain and weight loss	**All-cause mortality**: Weight loss increased the risk by 45%<br>Weight gain was not associated<br><br>**Cancer mortality**: neither weight loss nor weight gain was associated<br><br>**CVD mortality**: Weight gain increased the risk by 21%· Weight loss was not associated	High heterogeneity between the included studies<br><br>Most studies examined weight change and, in a few studies, change in BMI was examined·
	dwelling adults aged 65 years	Risques, Incidence et Traitement (ESPRIT) study	face-to-face interviews·	with an increased mortality risk after adjusting for health and lifestyle factors· In men, a baseline self-reported recent weight loss of >3 kg was associated with a 52% increase in mortality risk	

© 2023 Hussain SM et al. *JAMA Network Open.*

Author, Year	Study population	Study name	Exposure	Findings related to mortality	Comments
Systematic reviews					
Alharbi 2021[3]	30 studies, including participants aged >65 years Studies published before June 2020 were included	N/A	Weight gain, weight loss, weight fluctuation	**All-cause mortality**: Based on 30 studies weight loss was associated with a 59% increase risk· Based on 27 studies weight gain was associated with a 10% increase risk·<br>Based on 4 studies weight fluctuation was associated with a 63% increased risk·	Only in 6 studies change in BMI was examined· In the rest of the studies change in weight (kg) was examined·<br><br>High heterogeneity between the included studies
Karahalios 2017[9]	26 studies including participants aged 40-65 years·<br>Studies were published before September 2015	N/A	Weight gain and weight loss	**All-cause mortality**: Weight loss increased the risk by 45%<br>Weight gain was not associated<br><br>**Cancer mortality**: neither weight loss nor weight gain was associated<br><br>**CVD mortality**: Weight gain increased the risk by 21%· Weight loss was not associated	High heterogeneity between the included studies<br><br>Most studies examined weight change and, in a few studies, change in BMI was examined·
Okada 2021[10]	69,681 participants aged 40-79 years	Japan Collaborative Cohort Study for Evaluation of Cancer Risk	Weight change in kg calculated from recalled weight at age 20 and measured weight at baseline· 11 weight change categories: weight loss >12·5	**CVD mortality**: Any weight loss was associated with increased risk; only >12·5 kg weight gain was associated with increased risk	People in the weight loss group were older than those in the weight gain group· For example, mean age of weight loss >12·5 kg vs weight gain >12·5 kg was<br>65·5 vs 54·6 years

© 2023 Hussain SM et al. *JAMA Network Open.*

Author, Year	Study population	Study name	Exposure	Findings related to mortality	Comments
Systematic reviews					
Alharbi 2021[3]	30 studies, including participants aged >65 years Studies published before June 2020 were included	N/A	Weight gain, weight loss, weight fluctuation	**All-cause mortality**: Based on 30 studies weight loss was associated with a 59% increase risk· Based on 27 studies weight gain was associated with a 10% increase risk·<br>Based on 4 studies weight fluctuation was associated with a 63% increased risk·	Only in 6 studies change in BMI was examined· In the rest of the studies change in weight (kg) was examined·<br><br>High heterogeneity between the included studies
Karahalios 2017[9]	26 studies including participants aged 40-65 years·<br>Studies were published before September 2015	N/A	Weight gain and weight loss	**All-cause mortality**: Weight loss increased the risk by 45%<br>Weight gain was not associated<br><br>**Cancer mortality**: neither weight loss nor weight gain was associated<br><br>**CVD mortality**: Weight gain increased the risk by 21%· Weight loss was not associated	High heterogeneity between the included studies<br><br>Most studies examined weight change and, in a few studies, change in BMI was examined·
			kg, 10-12·4 kg, 7·5 to 9·9 kg, 5-7·4 kg, 2·5-4·9 kg;<br>Stable weight within 2·4 kg change;<br>Weight gain >12·5 kg, 10-12·4 kg, 7·5		

© 2023 Hussain SM et al. *JAMA Network Open.*

Author, Year	Study population	Study name	Exposure	Findings related to mortality	Comments
Systematic reviews					
Alharbi 2021[3]	30 studies, including participants aged >65 years Studies published before June 2020 were included	N/A	Weight gain, weight loss, weight fluctuation	**All-cause mortality**: Based on 30 studies weight loss was associated with a 59% increase risk· Based on 27 studies weight gain was associated with a 10% increase risk·<br>Based on 4 studies weight fluctuation was associated with a 63% increased risk·	Only in 6 studies change in BMI was examined· In the rest of the studies change in weight (kg) was examined·<br><br>High heterogeneity between the included studies
Karahalios 2017[9]	26 studies including participants aged 40-65 years·<br>Studies were published before September 2015	N/A	Weight gain and weight loss	**All-cause mortality**: Weight loss increased the risk by 45%<br>Weight gain was not associated<br><br>**Cancer mortality**: neither weight loss nor weight gain was associated<br><br>**CVD mortality**: Weight gain increased the risk by 21%· Weight loss was not associated	High heterogeneity between the included studies<br><br>Most studies examined weight change and, in a few studies, change in BMI was examined·
			to 9·9 kg, 5-7·4 kg, 2·5-4·9 kg		
Suh 2021[11]	10,254 participants aged >45 years	Korean Longitudinal Study of Aging	Weight change from 2006 to 2008· 3 weight change categories: within 5 kg weight change (‘stable’,	**All-cause mortality**: >5 kg weight loss increased the risk by 62% in participants aged 45-65 years, and by 56% in participants older than 65	

© 2023 Hussain SM et al. *JAMA Network Open.*

Author, Year	Study population	Study name	Exposure	Findings related to mortality	Comments
Systematic reviews					
Alharbi 2021[3]	30 studies, including participants aged >65 years Studies published before June 2020 were included	N/A	Weight gain, weight loss, weight fluctuation	**All-cause mortality**: Based on 30 studies weight loss was associated with a 59% increase risk· Based on 27 studies weight gain was associated with a 10% increase risk·<br>Based on 4 studies weight fluctuation was associated with a 63% increased risk·	Only in 6 studies change in BMI was examined· In the rest of the studies change in weight (kg) was examined·<br><br>High heterogeneity between the included studies
Karahalios 2017[9]	26 studies including participants aged 40-65 years·<br>Studies were published before September 2015	N/A	Weight gain and weight loss	**All-cause mortality**: Weight loss increased the risk by 45%<br>Weight gain was not associated<br><br>**Cancer mortality**: neither weight loss nor weight gain was associated<br><br>**CVD mortality**: Weight gain increased the risk by 21%· Weight loss was not associated	High heterogeneity between the included studies<br><br>Most studies examined weight change and, in a few studies, change in BMI was examined·
			reference category), weight gain >5 kg, and weight loss >5 kg·	years· Weight gain was not associated with the risk	
Son 2020[12]	100256 participants aged >65 years	Data from the Korean National Health	Weight change over 4 years· 3 weight categories: weight loss >5%,	**All-cause mortality**: Weight loss increased the rate by 68%, whereas weight gain increased the rate by 10%·	In subgroup analysis, for participants who were underweight at baseline,

© 2023 Hussain SM et al. *JAMA Network Open.*

Author, Year	Study population	Study name	Exposure	Findings related to mortality	Comments
Systematic reviews					
Alharbi 2021[3]	30 studies, including participants aged >65 years Studies published before June 2020 were included	N/A	Weight gain, weight loss, weight fluctuation	**All-cause mortality**: Based on 30 studies weight loss was associated with a 59% increase risk· Based on 27 studies weight gain was associated with a 10% increase risk·<br>Based on 4 studies weight fluctuation was associated with a 63% increased risk·	Only in 6 studies change in BMI was examined· In the rest of the studies change in weight (kg) was examined·<br><br>High heterogeneity between the included studies
Karahalios 2017[9]	26 studies including participants aged 40-65 years·<br>Studies were published before September 2015	N/A	Weight gain and weight loss	**All-cause mortality**: Weight loss increased the risk by 45%<br>Weight gain was not associated<br><br>**Cancer mortality**: neither weight loss nor weight gain was associated<br><br>**CVD mortality**: Weight gain increased the risk by 21%· Weight loss was not associated	High heterogeneity between the included studies<br><br>Most studies examined weight change and, in a few studies, change in BMI was examined·
		Insurance Corporation records	stable weight within 5% weight change, weight gain >5%		current cigarette smokers or heavy alcohol drinkers, weight gain did not increase the mortality rate·
Huang 2020[13]	17773 participants aged >50 years	Guangzhou Biobank Cohort Study	Change in BMI and WC were calculated between 2003-8 and 2008-	**All-cause mortality**:<br>>5% BMI loss and >5% WC loss increased the risk	

© 2023 Hussain SM et al. *JAMA Network Open.*

Author, Year	Study population	Study name	Exposure	Findings related to mortality	Comments
Systematic reviews					
Alharbi 2021[3]	30 studies, including participants aged >65 years Studies published before June 2020 were included	N/A	Weight gain, weight loss, weight fluctuation	**All-cause mortality**: Based on 30 studies weight loss was associated with a 59% increase risk· Based on 27 studies weight gain was associated with a 10% increase risk·<br>Based on 4 studies weight fluctuation was associated with a 63% increased risk·	Only in 6 studies change in BMI was examined· In the rest of the studies change in weight (kg) was examined·<br><br>High heterogeneity between the included studies
Karahalios 2017[9]	26 studies including participants aged 40-65 years·<br>Studies were published before September 2015	N/A	Weight gain and weight loss	**All-cause mortality**: Weight loss increased the risk by 45%<br>Weight gain was not associated<br><br>**Cancer mortality**: neither weight loss nor weight gain was associated<br><br>**CVD mortality**: Weight gain increased the risk by 21%· Weight loss was not associated	High heterogeneity between the included studies<br><br>Most studies examined weight change and, in a few studies, change in BMI was examined·
			12 surveys· 3 groups: >5% BMI loss, within 5% BMI change (stable), and >5% BMI gain· Similar classification	**CVD mortality**: >5% BMI loss and >5% WC loss increased the risk<br>**Cancer mortality**: >5% decrease in BMI increased the risk· No association for WC was observed	

© 2023 Hussain SM et al. *JAMA Network Open.*

Author, Year	Study population	Study name	Exposure	Findings related to mortality	Comments
Systematic reviews					
Alharbi 2021[3]	30 studies, including participants aged >65 years Studies published before June 2020 were included	N/A	Weight gain, weight loss, weight fluctuation	**All-cause mortality**: Based on 30 studies weight loss was associated with a 59% increase risk· Based on 27 studies weight gain was associated with a 10% increase risk·<br>Based on 4 studies weight fluctuation was associated with a 63% increased risk·	Only in 6 studies change in BMI was examined· In the rest of the studies change in weight (kg) was examined·<br><br>High heterogeneity between the included studies
Karahalios 2017[9]	26 studies including participants aged 40-65 years·<br>Studies were published before September 2015	N/A	Weight gain and weight loss	**All-cause mortality**: Weight loss increased the risk by 45%<br>Weight gain was not associated<br><br>**Cancer mortality**: neither weight loss nor weight gain was associated<br><br>**CVD mortality**: Weight gain increased the risk by 21%· Weight loss was not associated	High heterogeneity between the included studies<br><br>Most studies examined weight change and, in a few studies, change in BMI was examined·
			applied for WC change		
Nishida 2019[14]	1229 community-dwelling older adults<br>aged >65 years		Weight data was extracted from health check-up records of 2011 and 2013· 3 categories: >4·8%	**All-cause mortality**<br>Only weight loss was associated with increased risk·	

© 2023 Hussain SM et al. *JAMA Network Open.*

Author, Year	Study population	Study name	Exposure	Findings related to mortality	Comments
Systematic reviews					
Alharbi 2021[3]	30 studies, including participants aged >65 years Studies published before June 2020 were included	N/A	Weight gain, weight loss, weight fluctuation	**All-cause mortality**: Based on 30 studies weight loss was associated with a 59% increase risk· Based on 27 studies weight gain was associated with a 10% increase risk·<br>Based on 4 studies weight fluctuation was associated with a 63% increased risk·	Only in 6 studies change in BMI was examined· In the rest of the studies change in weight (kg) was examined·<br><br>High heterogeneity between the included studies
Karahalios 2017[9]	26 studies including participants aged 40-65 years·<br>Studies were published before September 2015	N/A	Weight gain and weight loss	**All-cause mortality**: Weight loss increased the risk by 45%<br>Weight gain was not associated<br><br>**Cancer mortality**: neither weight loss nor weight gain was associated<br><br>**CVD mortality**: Weight gain increased the risk by 21%· Weight loss was not associated	High heterogeneity between the included studies<br><br>Most studies examined weight change and, in a few studies, change in BMI was examined·
			weight loss, stable weight (between a 4·8% decrease and a 3·1% increase), >3·1% weight gain		
Chen 2019[15]	36051 participants	NHANES survey	Weight change between 10 years	**All-cause mortality**: weight loss and >20 kg weight gain	Participants in the obese to non-obese group was older than those

© 2023 Hussain SM et al. *JAMA Network Open.*

Author, Year	Study population	Study name	Exposure	Findings related to mortality	Comments
Systematic reviews					
Alharbi 2021[3]	30 studies, including participants aged >65 years Studies published before June 2020 were included	N/A	Weight gain, weight loss, weight fluctuation	**All-cause mortality**: Based on 30 studies weight loss was associated with a 59% increase risk· Based on 27 studies weight gain was associated with a 10% increase risk·<br>Based on 4 studies weight fluctuation was associated with a 63% increased risk·	Only in 6 studies change in BMI was examined· In the rest of the studies change in weight (kg) was examined·<br><br>High heterogeneity between the included studies
Karahalios 2017[9]	26 studies including participants aged 40-65 years·<br>Studies were published before September 2015	N/A	Weight gain and weight loss	**All-cause mortality**: Weight loss increased the risk by 45%<br>Weight gain was not associated<br><br>**Cancer mortality**: neither weight loss nor weight gain was associated<br><br>**CVD mortality**: Weight gain increased the risk by 21%· Weight loss was not associated	High heterogeneity between the included studies<br><br>Most studies examined weight change and, in a few studies, change in BMI was examined·
	aged >=40 years		before baseline (recalled weight) and baseline (measured weight)· 5 weight change categories: Weight loss >2·5kg, Stable	was related with increased risk, xx%?<br><br>**CVD mortality**: An obese to non-obese weight change pattern from middle to late adulthood was associated with increased risk·	who were stable normal or stable obesity category<br>Mean (95% CI) age<br>59·9 (58·3 to 61·5) vs 56·8 (56·2 to 57·4) vs 53·5 (52·8 to 54·2)

© 2023 Hussain SM et al. *JAMA Network Open.*

Author, Year	Study population	Study name	Exposure	Findings related to mortality	Comments
Systematic reviews					
Alharbi 2021[3]	30 studies, including participants aged >65 years Studies published before June 2020 were included	N/A	Weight gain, weight loss, weight fluctuation	**All-cause mortality**: Based on 30 studies weight loss was associated with a 59% increase risk· Based on 27 studies weight gain was associated with a 10% increase risk·<br>Based on 4 studies weight fluctuation was associated with a 63% increased risk·	Only in 6 studies change in BMI was examined· In the rest of the studies change in weight (kg) was examined·<br><br>High heterogeneity between the included studies
Karahalios 2017[9]	26 studies including participants aged 40-65 years·<br>Studies were published before September 2015	N/A	Weight gain and weight loss	**All-cause mortality**: Weight loss increased the risk by 45%<br>Weight gain was not associated<br><br>**Cancer mortality**: neither weight loss nor weight gain was associated<br><br>**CVD mortality**: Weight gain increased the risk by 21%· Weight loss was not associated	High heterogeneity between the included studies<br><br>Most studies examined weight change and, in a few studies, change in BMI was examined·
			weight within 2·5 kg change (reference)<br>Weight gain >2·5-10 kg, >10-20 kg, >20 kg<br>BMI categories: stable normal		

© 2023 Hussain SM et al. *JAMA Network Open.*

Author, Year	Study population	Study name	Exposure	Findings related to mortality	Comments
Systematic reviews					
Alharbi 2021[3]	30 studies, including participants aged >65 years Studies published before June 2020 were included	N/A	Weight gain, weight loss, weight fluctuation	**All-cause mortality**: Based on 30 studies weight loss was associated with a 59% increase risk· Based on 27 studies weight gain was associated with a 10% increase risk·<br>Based on 4 studies weight fluctuation was associated with a 63% increased risk·	Only in 6 studies change in BMI was examined· In the rest of the studies change in weight (kg) was examined·<br><br>High heterogeneity between the included studies
Karahalios 2017[9]	26 studies including participants aged 40-65 years·<br>Studies were published before September 2015	N/A	Weight gain and weight loss	**All-cause mortality**: Weight loss increased the risk by 45%<br>Weight gain was not associated<br><br>**Cancer mortality**: neither weight loss nor weight gain was associated<br><br>**CVD mortality**: Weight gain increased the risk by 21%· Weight loss was not associated	High heterogeneity between the included studies<br><br>Most studies examined weight change and, in a few studies, change in BMI was examined·
			(<25·0 kg/m^2 at both times), maximum overweight (25·0-29·9 kg/m^2<br>at either time but not ≥30·0 at the other time), obese		

© 2023 Hussain SM et al. *JAMA Network Open.*

Author, Year	Study population	Study name	Exposure	Findings related to mortality	Comments
Systematic reviews					
Alharbi 2021[3]	30 studies, including participants aged >65 years Studies published before June 2020 were included	N/A	Weight gain, weight loss, weight fluctuation	**All-cause mortality**: Based on 30 studies weight loss was associated with a 59% increase risk· Based on 27 studies weight gain was associated with a 10% increase risk·<br>Based on 4 studies weight fluctuation was associated with a 63% increased risk·	Only in 6 studies change in BMI was examined· In the rest of the studies change in weight (kg) was examined·<br><br>High heterogeneity between the included studies
Karahalios 2017[9]	26 studies including participants aged 40-65 years·<br>Studies were published before September 2015	N/A	Weight gain and weight loss	**All-cause mortality**: Weight loss increased the risk by 45%<br>Weight gain was not associated<br><br>**Cancer mortality**: neither weight loss nor weight gain was associated<br><br>**CVD mortality**: Weight gain increased the risk by 21%· Weight loss was not associated	High heterogeneity between the included studies<br><br>Most studies examined weight change and, in a few studies, change in BMI was examined·
			to non-obese (≥30·0 at younger age and <30·0 later), non-obese to obese (<30·0 at younger age and ≥30·0 later), and stable obesity		

© 2023 Hussain SM et al. *JAMA Network Open.*

Author, Year	Study population	Study name	Exposure	Findings related to mortality	Comments
Systematic reviews					
Alharbi 2021[3]	30 studies, including participants aged >65 years Studies published before June 2020 were included	N/A	Weight gain, weight loss, weight fluctuation	**All-cause mortality**: Based on 30 studies weight loss was associated with a 59% increase risk· Based on 27 studies weight gain was associated with a 10% increase risk·<br>Based on 4 studies weight fluctuation was associated with a 63% increased risk·	Only in 6 studies change in BMI was examined· In the rest of the studies change in weight (kg) was examined·<br><br>High heterogeneity between the included studies
Karahalios 2017[9]	26 studies including participants aged 40-65 years·<br>Studies were published before September 2015	N/A	Weight gain and weight loss	**All-cause mortality**: Weight loss increased the risk by 45%<br>Weight gain was not associated<br><br>**Cancer mortality**: neither weight loss nor weight gain was associated<br><br>**CVD mortality**: Weight gain increased the risk by 21%· Weight loss was not associated	High heterogeneity between the included studies<br><br>Most studies examined weight change and, in a few studies, change in BMI was examined·
			(≥30·0 at both times)·		
Park 2018[16]	63,040 participants aged 45-75 years	The Multiethnic Cohort Study 7·3 years	Weight change from baseline to 10 years, 7 categories:	**All-cause mortality**: any weight loss and >10kg weight gain increased the risk	Data for all-cause mortality for the 65-75 years old people was extractable·

© 2023 Hussain SM et al. *JAMA Network Open.*

Author, Year	Study population	Study name	Exposure	Findings related to mortality	Comments
Systematic reviews					
Alharbi 2021[3]	30 studies, including participants aged >65 years Studies published before June 2020 were included	N/A	Weight gain, weight loss, weight fluctuation	**All-cause mortality**: Based on 30 studies weight loss was associated with a 59% increase risk· Based on 27 studies weight gain was associated with a 10% increase risk·<br>Based on 4 studies weight fluctuation was associated with a 63% increased risk·	Only in 6 studies change in BMI was examined· In the rest of the studies change in weight (kg) was examined·<br><br>High heterogeneity between the included studies
Karahalios 2017[9]	26 studies including participants aged 40-65 years·<br>Studies were published before September 2015	N/A	Weight gain and weight loss	**All-cause mortality**: Weight loss increased the risk by 45%<br>Weight gain was not associated<br><br>**Cancer mortality**: neither weight loss nor weight gain was associated<br><br>**CVD mortality**: Weight gain increased the risk by 21%· Weight loss was not associated	High heterogeneity between the included studies<br><br>Most studies examined weight change and, in a few studies, change in BMI was examined·
			Weight loss: >10 kg, >5-10 kg, >2·5-5 kg,<br>Stable weight ± 2·5 kg (reference)<br>Weight gain >2·5-5 kg, >5-10 kg, >10 kg	**CVD mortality**: any weight loss and >10kg weight gain increased the risk<br><br>**Cancer mortality**: any weight loss but no weight gain was associated with increased risk	People who gained weight were relatively younger than people who lost weight<br>Mean (SD) age of participants with 10 kg weight loss vs 10 kg weight gain: 59·6 ± 8·5 vs 52·7 ± 6·6

© 2023 Hussain SM et al. *JAMA Network Open.*

Author, Year	Study population	Study name	Exposure	Findings related to mortality	Comments
Systematic reviews					
Alharbi 2021[3]	30 studies, including participants aged >65 years Studies published before June 2020 were included	N/A	Weight gain, weight loss, weight fluctuation	**All-cause mortality**: Based on 30 studies weight loss was associated with a 59% increase risk· Based on 27 studies weight gain was associated with a 10% increase risk·<br>Based on 4 studies weight fluctuation was associated with a 63% increased risk·	Only in 6 studies change in BMI was examined· In the rest of the studies change in weight (kg) was examined·<br><br>High heterogeneity between the included studies
Karahalios 2017[9]	26 studies including participants aged 40-65 years·<br>Studies were published before September 2015	N/A	Weight gain and weight loss	**All-cause mortality**: Weight loss increased the risk by 45%<br>Weight gain was not associated<br><br>**Cancer mortality**: neither weight loss nor weight gain was associated<br><br>**CVD mortality**: Weight gain increased the risk by 21%· Weight loss was not associated	High heterogeneity between the included studies<br><br>Most studies examined weight change and, in a few studies, change in BMI was examined·
Mulligan 2018[17]	12,580 participants, aged 39-78 years	European Prospective Investigation into Cancer in Norfolk (EPIC-Norfolk) cohort Study	Weight change calculated between 1993-1997 and 1998-2000 surveys· 5 categories: weight loss >5kg, 2·5-5 kg, stable weight	***Men***<br>**All-cause mortality**: any weight loss increased risk<br>**Cancer mortality**: no association<br>**CVD mortality**: any weight loss increased risk	Participants in the weight loss group were older than weight gain group· For example, mean (SD) age of weight loss >5 kg vs weight gain >5 kg was 60·5 (9·2) vs 55·9 (8·7)

© 2023 Hussain SM et al. *JAMA Network Open.*

Author, Year	Study population	Study name	Exposure	Findings related to mortality	Comments
Systematic reviews					
Alharbi 2021[3]	30 studies, including participants aged >65 years Studies published before June 2020 were included	N/A	Weight gain, weight loss, weight fluctuation	**All-cause mortality**: Based on 30 studies weight loss was associated with a 59% increase risk· Based on 27 studies weight gain was associated with a 10% increase risk·<br>Based on 4 studies weight fluctuation was associated with a 63% increased risk·	Only in 6 studies change in BMI was examined· In the rest of the studies change in weight (kg) was examined·<br><br>High heterogeneity between the included studies
Karahalios 2017[9]	26 studies including participants aged 40-65 years·<br>Studies were published before September 2015	N/A	Weight gain and weight loss	**All-cause mortality**: Weight loss increased the risk by 45%<br>Weight gain was not associated<br><br>**Cancer mortality**: neither weight loss nor weight gain was associated<br><br>**CVD mortality**: Weight gain increased the risk by 21%· Weight loss was not associated	High heterogeneity between the included studies<br><br>Most studies examined weight change and, in a few studies, change in BMI was examined·
			within 2·5 kg, weight gain 2·5-5 kg, >5 kg	**Respiratory mortality**:<br>only >5 kg weight loss and any weight gain increased the risk<br><br>***Women***<br>**All=cause mortality**: any weight loss increased the risk	

© 2023 Hussain SM et al. *JAMA Network Open.*

Author, Year	Study population	Study name	Exposure	Findings related to mortality	Comments
Systematic reviews					
Alharbi 2021[3]	30 studies, including participants aged >65 years Studies published before June 2020 were included	N/A	Weight gain, weight loss, weight fluctuation	**All-cause mortality**: Based on 30 studies weight loss was associated with a 59% increase risk· Based on 27 studies weight gain was associated with a 10% increase risk·<br>Based on 4 studies weight fluctuation was associated with a 63% increased risk·	Only in 6 studies change in BMI was examined· In the rest of the studies change in weight (kg) was examined·<br><br>High heterogeneity between the included studies
Karahalios 2017[9]	26 studies including participants aged 40-65 years·<br>Studies were published before September 2015	N/A	Weight gain and weight loss	**All-cause mortality**: Weight loss increased the risk by 45%<br>Weight gain was not associated<br><br>**Cancer mortality**: neither weight loss nor weight gain was associated<br><br>**CVD mortality**: Weight gain increased the risk by 21%· Weight loss was not associated	High heterogeneity between the included studies<br><br>Most studies examined weight change and, in a few studies, change in BMI was examined·
				**Cancer mortality**: no association<br>**CVD mortality**: any weight loss increased the risk<br>**Respiratory mortality**: only >5 kg weight loss increased the risk	

© 2023 Hussain SM et al. *JAMA Network Open.*

Author, Year	Study population	Study name	Exposure	Findings related to mortality	Comments
Systematic reviews					
Alharbi 2021[3]	30 studies, including participants aged >65 years Studies published before June 2020 were included	N/A	Weight gain, weight loss, weight fluctuation	**All-cause mortality**: Based on 30 studies weight loss was associated with a 59% increase risk· Based on 27 studies weight gain was associated with a 10% increase risk·<br>Based on 4 studies weight fluctuation was associated with a 63% increased risk·	Only in 6 studies change in BMI was examined· In the rest of the studies change in weight (kg) was examined·<br><br>High heterogeneity between the included studies
Karahalios 2017[9]	26 studies including participants aged 40-65 years·<br>Studies were published before September 2015	N/A	Weight gain and weight loss	**All-cause mortality**: Weight loss increased the risk by 45%<br>Weight gain was not associated<br><br>**Cancer mortality**: neither weight loss nor weight gain was associated<br><br>**CVD mortality**: Weight gain increased the risk by 21%· Weight loss was not associated	High heterogeneity between the included studies<br><br>Most studies examined weight change and, in a few studies, change in BMI was examined·
Pan 2018[18]	36338 participants aged 45-74 years	Singapore Chinese Health Study	Weight change calculated as the difference between 1993-1998 and 1999–2004 surveys· 5 categories: weight loss ≥10%, 5·1–	**All-cause mortality**: any weight loss and >10% weight gain increased the risk<br>**Cancer mortality**: no association<br>**CVD mortality**: >10% weight loss and >10%	Magnitude of weight loss was greater than that of weight gain

© 2023 Hussain SM et al. *JAMA Network Open.*

Author, Year	Study population	Study name	Exposure	Findings related to mortality	Comments
Systematic reviews					
Alharbi 2021[3]	30 studies, including participants aged >65 years Studies published before June 2020 were included	N/A	Weight gain, weight loss, weight fluctuation	**All-cause mortality**: Based on 30 studies weight loss was associated with a 59% increase risk· Based on 27 studies weight gain was associated with a 10% increase risk·<br>Based on 4 studies weight fluctuation was associated with a 63% increased risk·	Only in 6 studies change in BMI was examined· In the rest of the studies change in weight (kg) was examined·<br><br>High heterogeneity between the included studies
Karahalios 2017[9]	26 studies including participants aged 40-65 years·<br>Studies were published before September 2015	N/A	Weight gain and weight loss	**All-cause mortality**: Weight loss increased the risk by 45%<br>Weight gain was not associated<br><br>**Cancer mortality**: neither weight loss nor weight gain was associated<br><br>**CVD mortality**: Weight gain increased the risk by 21%· Weight loss was not associated	High heterogeneity between the included studies<br><br>Most studies examined weight change and, in a few studies, change in BMI was examined·
			9·9%, stable weight<br>±5%, weight gain 5·1–9·9% and ≥10%	weight gain increased the risk<br>**Respiratory mortality**: only >10% weight loss increased the risk	

© 2023 Hussain SM et al. *JAMA Network Open.*

Author, Year	Study population	Study name	Exposure	Findings related to mortality	Comments
Systematic reviews					
Alharbi 2021[3]	30 studies, including participants aged >65 years Studies published before June 2020 were included	N/A	Weight gain, weight loss, weight fluctuation	**All-cause mortality**: Based on 30 studies weight loss was associated with a 59% increase risk· Based on 27 studies weight gain was associated with a 10% increase risk·<br>Based on 4 studies weight fluctuation was associated with a 63% increased risk·	Only in 6 studies change in BMI was examined· In the rest of the studies change in weight (kg) was examined·<br><br>High heterogeneity between the included studies
Karahalios 2017[9]	26 studies including participants aged 40-65 years·<br>Studies were published before September 2015	N/A	Weight gain and weight loss	**All-cause mortality**: Weight loss increased the risk by 45%<br>Weight gain was not associated<br><br>**Cancer mortality**: neither weight loss nor weight gain was associated<br><br>**CVD mortality**: Weight gain increased the risk by 21%· Weight loss was not associated	High heterogeneity between the included studies<br><br>Most studies examined weight change and, in a few studies, change in BMI was examined·
Haugsgjerd 2017[19]	2935 participants aged 71-74 years	The Hordaland Health Study	Weight change measures between 1992-93 and 1997-99 surveys· 3 categories: weight loss<br>(≥5%), stable weight (±<5%),	**All-cause mortality**: Weight lost ≥5% increased the risk; weight gain ≥5% did not·	

© 2023 Hussain SM et al. *JAMA Network Open.*

Author, Year	Study population	Study name	Exposure	Findings related to mortality	Comments
Systematic reviews					
Alharbi 2021[3]	30 studies, including participants aged >65 years Studies published before June 2020 were included	N/A	Weight gain, weight loss, weight fluctuation	**All-cause mortality**: Based on 30 studies weight loss was associated with a 59% increase risk· Based on 27 studies weight gain was associated with a 10% increase risk·<br>Based on 4 studies weight fluctuation was associated with a 63% increased risk·	Only in 6 studies change in BMI was examined· In the rest of the studies change in weight (kg) was examined·<br><br>High heterogeneity between the included studies
Karahalios 2017[9]	26 studies including participants aged 40-65 years·<br>Studies were published before September 2015	N/A	Weight gain and weight loss	**All-cause mortality**: Weight loss increased the risk by 45%<br>Weight gain was not associated<br><br>**Cancer mortality**: neither weight loss nor weight gain was associated<br><br>**CVD mortality**: Weight gain increased the risk by 21%· Weight loss was not associated	High heterogeneity between the included studies<br><br>Most studies examined weight change and, in a few studies, change in BMI was examined·
			and weight gain (≥5%)		
Beleigoli 2017[20]	1138 participants aged >60 years	The Bambuí (Brazil) Cohort Study of Aging	Weight and WC were assessed at baseline and three years later· 3 categories each for weight and WC:	**All-cause mortality**: Weight loss increased the risk by 69% whereas weight gain increased the risk by 37% except in those who were physically active in which	

© 2023 Hussain SM et al. *JAMA Network Open.*

Author, Year	Study population	Study name	Exposure	Findings related to mortality	Comments
Systematic reviews					
Alharbi 2021[3]	30 studies, including participants aged >65 years Studies published before June 2020 were included	N/A	Weight gain, weight loss, weight fluctuation	**All-cause mortality**: Based on 30 studies weight loss was associated with a 59% increase risk· Based on 27 studies weight gain was associated with a 10% increase risk·<br>Based on 4 studies weight fluctuation was associated with a 63% increased risk·	Only in 6 studies change in BMI was examined· In the rest of the studies change in weight (kg) was examined·<br><br>High heterogeneity between the included studies
Karahalios 2017[9]	26 studies including participants aged 40-65 years·<br>Studies were published before September 2015	N/A	Weight gain and weight loss	**All-cause mortality**: Weight loss increased the risk by 45%<br>Weight gain was not associated<br><br>**Cancer mortality**: neither weight loss nor weight gain was associated<br><br>**CVD mortality**: Weight gain increased the risk by 21%· Weight loss was not associated	High heterogeneity between the included studies<br><br>Most studies examined weight change and, in a few studies, change in BMI was examined·
			loss (>5% reduction), stable within 5% of baseline, gain (>5% increase) measure	weight gain was associated with decreased mortality·<br>Similar relationship was observed for WC change·	

BMI, body mass index; WC, waist circumference; CVD, cardiovascular disease

© 2023 Hussain SM et al. *JAMA Network Open.*
