# Supplementary material for: Associations of Change in Body Size With All-Cause and Cause-Specific Mortality Among Healthy Older Adults
Source: JAMA Netw Open. 2023 Apr 10;6(4):e237482. doi: 10.1001/jamanetworkopen.2023.7482 (PMC10087052; doi:10.1001/jamanetworkopen.2023.7482)
Supplement: Supplement 2. — Data Sharing Statement [file jamanetwopen-e237482-s002.pdf]

Data Sharing Statement

Hussain. Associations of Change in Body Size With All-Cause and Cause-Specific Mortality Among Healthy Older Adults. *JAMA Netw Open*. Published April 10, 2023. doi:10.1001/jamanetworkopen.2023.7482

Data
Data available: No

Additional Information
Explanation for why data not available: Data can be obtained after communicating the corresponding author
